# Peptide Vaccination Against PD-L1 With IO103 a Novel Immune Modulatory Vaccine in Multiple Myeloma: A Phase I First-in-Human Trial

**DOI:** 10.3389/fimmu.2020.595035

**Published:** 2020-11-09

**Authors:** Nicolai Grønne Jørgensen, Uffe Klausen, Jacob Handlos Grauslund, Carsten Helleberg, Thomas Granum Aagaard, Trung Hieu Do, Shamaila Munir Ahmad, Lars Rønn Olsen, Tobias Wirenfeldt Klausen, Marie Fredslund Breinholt, Morten Hansen, Evelina Martinenaite, Özcan Met, Inge Marie Svane, Lene Meldgaard Knudsen, Mads Hald Andersen

**Affiliations:** ^1^ National Center for Cancer Immune Therapy (CCIT-DK), Department of Oncology, Copenhagen University Hospital, Herlev, Denmark; ^2^ Department of Hematology, Copenhagen University Hospital, Herlev, Denmark; ^3^ Department of Health Technology, Technical University of Denmark, Kgs. Lyngby, Denmark; ^4^ Department of Pathology, Copenhagen University Hospital, Herlev, Denmark; ^5^ Department of Immunology and Microbiology, University of Copenhagen, Copenhagen, Denmark

**Keywords:** peptide, vaccination, PD-L1, first-in-human, myeloma

## Abstract

**Background:**

Immune checkpoint blockade with monoclonal antibodies targeting programmed death 1 (PD-1) and its ligand PD-L1 has played a major role in the rise of cancer immune therapy. We have identified naturally occurring self-reactive T cells specific to PD-L1 in both healthy donors and cancer patients. Stimulation with a PD-L1 peptide (IO103), activates these cells to exhibit inflammatory and anti-regulatory functions that include cytotoxicity against PD-L1–expressing target cells. This prompted the initiation of the present first-in-human study of vaccination with IO103, registered at clinicaltrials.org (NCT03042793).

**Methods:**

Ten patients with multiple myeloma who were up to 6 months after high dose chemotherapy with autologous stem cell support, were enrolled. Subcutaneous vaccinations with IO103 with the adjuvant Montanide ISA 51 was given up to fifteen times during 1 year. Safety was assessed by the common toxicity criteria for adverse events (CTCAE). Immunogenicity of the vaccine was evaluated using IFNγ enzyme linked immunospot and intracellular cytokine staining on blood and skin infiltrating lymphocytes from sites of delayed-type hypersensitivity. The clinical course was described.

**Results:**

All adverse reactions to the PD-L1 vaccine were below CTCAE grade 3, and most were grade 1–2 injection site reactions. The total rate of adverse events was as expected for the population. All patients exhibited peptide specific immune responses in peripheral blood mononuclear cells and in skin-infiltrating lymphocytes after a delayed-type hypersensitivity test. The clinical course was as expected for the population. Three of 10 patients had improvements of responses which coincided with the vaccinations.

**Conclusion:**

Vaccination against PD-L1 was associated with low toxicity and high immunogenicity. This study has prompted the initiation of later phase trials to assess the vaccines efficacy.

**Clinical Trial Registration:**

clinicaltrials.org, identifier NCT03042793.

## Introduction

The development of monoclonal antibodies that block immune checkpoint molecules (ICB) has launched a new era in the treatment of malignancies. However, ICB treatment benefits a minority of patients with cancer and is associated with side effects (1, 2). We have recently explored whether ICBs can also be targeted by peptide vaccination. Such a vaccine could potentially combine the low toxicity of vaccination with a therapy that mitigates cancer-imposed immune inhibition.

In preclinical studies, we have demonstrated that the ICB programmed death ligand 1 (PD-L1) is recognized by T cells in both healthy donors and cancer patients (3). These PD-L1–reactive T cells can be activated by peptide stimulation. From the signal peptide of PD-L1, we developed a highly immunogenic 19-amino-acid peptide, designated IO103. IO103-stimulated PD-L1–specific T cells are cytotoxic to cancer cell lines, including melanoma, renal cell carcinoma, breast cancer, leukemia, and chronic myeloproliferative neoplasms (4–7). *In vitro* stimulation with PD-L1 peptide boosted immune responses to a dendritic cell (DC) vaccine (8). Similarly, immune responses to PD-L1 have been observed in multiple myeloma (MM) (Jørgensen et al., in preparation).

In MM, T cells and natural killer (NK) cells in the tumor microenvironment exhibit upregulated PD-1, and MM cells, osteoclasts, and DCs are often PD-L1^+^ (9–16). The PD-1/PD-L1 pathway indirectly promotes myeloma progression by causing failure of immune control. Moreover, bone marrow (BM) stromal cells induce myeloma cells to express PD-L1, inducing increased tumor cell proliferation and reduced susceptibility to chemotherapy (17). PD-L1 expression is often detected on plasma cells in extramedullary plasmacytomas of late-stage disease (18). Furthermore, PD-1 levels on T cells in myeloma patients are negatively correlated with survival (19), and PD-L1 upregulation on MM cells is common among patients with relapsed or refractory MM and associated with an aggressive phenotype (20). However, PD-1 blockade does not exhibit single-agent activity in MM (21), and initial promising data regarding combination therapy with PD-1 ICB and immunomodulatory drugs with dexamethasone was not confirmed in randomized trials (22, 23). The present study was initiated before these randomized trials were halted by the FDA in 2017.

Peptide vaccination has shown promising results in early-stage neoplasia, and combined with chemotherapy (24). High-dose chemotherapy with autologous stem cell support (HDT)-mediated lymphodepletion yields a decreased Treg/CD8^+^ ratio, which theoretically should favor immunotherapy post-HDT (25, 26). Furthermore, in preclinical studies, homeostatic cytokine-driven peripheral T-cell expansion after lymphodepletion reportedly aids the establishment of antitumor responses to vaccines, prompting several studies of post-HDT immune therapy (27, 28). Hence, we chose to vaccinate patients as they were in post-HDT remission.

Based on previous studies of therapeutic cancer vaccines, we expected a low level of adverse reactions. The impressive preclinical immunogenicity of the peptide led us to expect the induction of strong immune responses to IO103. Here, we present the results from a phase I first-in-human study of subcutaneous vaccination with IO103 emulsified with the adjuvant Montanide.

## Subjects and Methods

### Study Design

In this first-in-human open-label single-armed study, the safety and immunogenicity of vaccinations using the PD-L1 peptide IO103 with the adjuvant Montanide was evaluated. Patients were enrolled at Herlev and Gentofte University Hospital, Copenhagen, Denmark, between February and November of 2017. The study was conducted in accordance with the Helsinki Declaration, and Good Clinical Practice (GCP) recommendations. All participants gave written informed consent before enrollment. The protocol was approved by the Ethics Committee of the Capital Region of Denmark, the National Board of Health, and the Danish Data Protection Agency, and registered at www.clinicaltrials.gov (NCT03042793; date of registration: February 2, 2017). Blood samples were obtained from a reference cohort of unvaccinated patients (*n* = 6) with MM and who concurrently received the same standard-of-care treatment as the vaccinated patients, after they gave written informed consent in an observational study approved by the Ethics Committee of the Capital Region of Denmark (Approval no. H-17010084). In the same study, serum was sampled from patients with smoldering multiple myeloma (n = 10).

With no previous human exposure to this vaccine to perform statistical power calculations, a sample size of 10 individuals was chosen based on experience from similar studies. Eligibility criteria included Eastern Cooperative Oncology Group performance status of ≤2, no severe comorbidities or autoimmune diseases, and no signs of myeloma relapse. Cytogenetic analyses were not required. **Supplementary Table 1** presents full inclusion and exclusion criteria. Patients with MM were enrolled to receive vaccination once they were in remission, between 4 weeks to 6 months post-HDT. No additional maintenance treatment was given. All patients received zoledronic acid to minimize the rate of skeletal osteolytic lesions.

### Treatment

Vaccination with a long peptide forces uptake and presentation by antigen presenting cells, whereas vaccination with short peptides (9–10 amino acids) could lead to merely coating HLA molecules. Thus, vaccination with long peptides permits inclusion of patients without HLA-type restriction, and is associated with stimulation of both CD4^+^ and CD8^+^ T cells (29). In this study, patients were administered subcutaneous vaccinations containing 100 μg IO103, a 19-amino-acid peptide (FMTYWHLLNAFTVTVPKDL) from the signal peptide of PD-L1 (PolyPeptide Laboratories, France). The peptide was dissolved in dimethylsulfoxide (DMSO), sterile filtered, and frozen at –20° Celsius (NUNC™ CryoTubes™ CryoLine System™ Internal Thread, Sigma-Aldrich). At ≤2 h before administration, the peptide was thawed and dissolved in sterile water for injection. Immediately before injection, the dissolved peptide was emulsified 1:1 with the adjuvant Montanide ISA-51 (Seppic Inc. Paris, France) to a total volume of 1 ml (30). Vaccinations were administered by subcutaneous injection every two weeks, repeated six times, and then once every 4 weeks until reaching a total of 15 vaccines.

### Clinical Evaluation

Adverse events were assessed according to CTCAE v.4.03. Patients were followed with frequent blood samples including a full myeloma panel and electrocardiograms. Clinical response was evaluated following International Myeloma Working Group (IMWG) response criteria (31). Time to next treatment was calculated from autologous stem cell transplant (ASCT) until initiation of next treatment.

### Blood and Bone Marrow Samples

Blood samples for isolation of serum and peripheral blood mononuclear cells (PBMCs) were obtained at baseline, after three vaccinations, after six vaccinations, and after 15 vaccinations or at relapse. Samples were kept at room temperature (RT) for ≤5 h until handling. PBMCs were isolated by gradient centrifugation of heparinized blood on Lymphoprep (STEMCELL Technologies) in LeucoSep tubes (Greiner Bio-One). Isolated PBMCs were cryopreserved in 90% human serum (Sigma-Aldrich) with 10% DMSO (Sigma-Aldrich) using controlled-rate freezing (Cool-Cell, Biocision) in a –80°C freezer. The next day, the ampules were transferred to –140°C. To obtain serum samples, blood was collected in 8-ml Vacuette gel tubes containing clot activator (Greiner Bio-One). The tubes were centrifuged, and serum was stored at –140°C. Serum and PBMCs were stored in 1.8-ml NUNC™ CryoTubes™ CryoLine System™ Internal Thread (Sigma-Aldrich).

Heparinized bone marrow samples (10 ml in a heparinized tube) were obtained at baseline, after six vaccines, and after 15 vaccines or at relapse. The samples were subjected to red blood cell lysis by adding Ortho-Lysing Buffer diluted 10× in H_2_0, followed by centrifugation and incubation for 15 min in the dark. The remaining cells were cryopreserved following the same procedure as for PBMCs.

### Delayed-Type Hypersensitivity and Skin-Infiltrating Lymphocytes

Presence of tumor-specific T cells in biopsies from delayed-type hypersensitivity (DTH) testing post-vaccination is correlated with clinical outcome (32). We assessed the presence of vaccine-reactive cells at DTH sites after six vaccinations. On the lower back, we performed three intradermal injections of IO103 without adjuvant and one control injection of aqueous solvent containing DMSO without peptide. At 48 h post-DTH injection, skin reaction was measured, and punch biopsies were taken from the sites of IO103-containing injections and cut into fragments. Fragments were cultured in 24-well plates for 3–5 weeks in RPMI-1640 with 10% human serum and 100 U/ml interleukin-2 (IL-2) with penicillin, streptomycin, and fungizone. Three times weekly, half the medium was replaced with fresh medium containing IL-2. Skin-infiltrating lymphocytes (SKILs) emigrated from the biopsies. After 3–5 weeks, SKILs were harvested and tested in ELISPOT assays (see below). The remaining SKILs were cryopreserved, as described for PBMCs.

### IFNγ ELISpot Assay

To assess T-cell responses against IO103, indirect interferon gamma Enzyme-Linked ImmunoSPOT (IFNγ-ELISpot) assays were performed as previously described (3). PBMCs were stimulated once *in vitro* to increase assay sensitivity (33). Briefly, cryopreserved PBMCs were thawed and stimulated once with IO103 at RT in 24-well plates with 0.5 ml X-VIVO medium. After 2 h, 1.5 ml X-VIVO medium with 5% human serum was added, and the plate was incubated at 37°C under 5% CO_2_. The next day, IL-2 was added, yielding a concentration of 120 U/ml. After 5–10 days, stimulated PBMCs were added to a 96-well nitrocellulose plate (MultiScreen, MAIP N45; Millipore) precoated with anti-IFNγ-mAb (mAb 1-DIK, Mabtech, Sweden). IO103 was added, and the cells were incubated overnight. Next day, the plates were washed, biotinylated secondary anti-INFγ mAb (Mabtech) was added, and the plates were incubated for 2 h at RT. Then, the plates were washed, Streptavidin-enzyme conjugate (AP-Avidin; Calbiochem/Invitrogen Life Technologies) was added, and the plates were incubated for 1 h at RT, and then washed again. Finally, the enzyme substrate NBT/BCIP (Invitrogen Life Technologies) was added, and the resulting spots were counted using the ImmunoSpot Series 2.0 Analyser (CTL Analyser). Maximum count was set to 500 spots/well. Raw data are available upon request.

IFNγ-ELISPOT assays on PBMCs were run in triplicate with 2.2–3.0 × 10^5^ cells/well. For graphic representation, numbers were normalized to 2.2 × 10^5^ cells/well. IFNγ-ELISPOT assays on SKILs were run in triplicate or quadruplicate with 3 × 10^5^ cells/well, using a reversed sequence of the IO103 peptide as a control. IFNγ-ELISPOT assays on BM samples were hampered by low viability and high background, but singlets were run from all time points from one patient.

### Flow Cytometry on PBMCs

Cryopreserved PBMCs were thawed in wash buffer (0.5% BSA, 2 mM EDTA in PBS) at 37°C, and Fc-receptors blocked by incubation with human IgG (20 mcg/ml). PBMCs were stained in three panels using the following antibodies: CD3-FITC, CD56-PE, CD11c-PE, CD8-PerCP, HLA-DR-PerCP, CD27-BV421, CD25-BV421, CD4-BV510, CD28-PE-Cy7, CD3-PE-Cy7, CD19-PE-Cy7, CD127-PE-Cy7, CD45RA-APC, CD56-BV510 (all from BD Bioscience, NJ, United States), CCR7-PE, PD-1-APC, CD14-BV421 (all from Biolegend, California, United States), CD16-FITC (Dako, Glostrup, Denmark), and NiR live-dead reagent for APC-Cy7 channel (Invitrogen-Thermo Fischer, United States). Two panels for analyzing regulatory T cells (Tregs) were run: one with intracellular FoxP3-PE and one with only surface markers, including CCR7-PE. Both Treg panels included CD45RA-FITC, CD4-PerCP, CD127-PE-Cy7, CCR4-APC, CD25-BV421, and CD15s-BV510. Samples were incubated with relevant antibodies for 20 min in the dark at 4°C, washed, and then analyzed on a FACS Canto II flow cytometer (BD) and analyzed using FACSDiva Software version 8.0.1 (BD). T cells in the CD4 and CD8 compartments were characterized by examining live singlet events in the PBMC (lymphocyte and monocyte) gate in the forward and side scatter plot. Naïve T cells were characterized as CCR7^+^CD45RA^+^, central memory (CM) as CCR7^+^CD45RA^–^, effector memory as CCR7^–^CD45RA^–^, and effector memory RA^+^ (EMRA) as CCR7^–^CD45RA^+^. Tregs were gated on PBMCs, singlets, live cells, and subsequently on CD4^+^ cells. **Supplementary Figure 10** presents the gating strategy for Tregs. Myeloid DCs (LIN^–^CD11c^+^HLA-DR^+^CD14^-^CD16^–^) and non-classical monocytes (LIN^–^CD11c^+^HLA-DR^+^CD14^–^CD16^+^) were gated from the Lineage (CD3/CD19/CD56) and CD14 negative and CD11c, HLA-DR positive fraction of PBMCs. To analyze SKILs’ cytokine secretion capability, intracellular cytokine staining was performed on SKILs incubated 5 h with or without 5 μg/ml IO103 (37°C, 5% CO_2_). GolgiPlug (BD) was applied before staining with CD3-APC-H7, CD4-PerCP/FITC, CD8-Pacific Blue/PerCP, and Horizon Fixable Viability Stain 510 (BD). The cells were fixed and permeabilized with Fixation/Permeabilization Buffer (eBioscience), following the manufacturer’s instructions, and then intracellularly stained using IFNγ-PE-Cy7/APC (eBioscience) and TNF-APC/BV421 (eBioscience). Relevant isotype controls were used to support correct compensation and confirm antibody specificity.

### Cytokines in Serum

Cytokines in serum samples were measured using the MSD Mesoscale V-Plex Human Cytokine 30-plex Kit (Catalog No. K15054D-1), following the manufacturer’s instructions, except that the samples were diluted four-fold instead of two-fold.

### Statistical Analysis

Responses in ELISpot assays were determined using the previously described distribution-free resampling (DFR) method as described by Moodie et al. (34). The Wilcoxon matched-pairs signed-rank test was used to compare responses to IO103 between baseline and later time points. For flow cytometry samples, the unpaired Mann-Whitney was used to compare lymphocyte subsets in vaccinated patients versus the reference cohort at an individual time point. As this exploratory analysis was descriptive and done *post hoc*, no formal multiple testing corrections were performed. *p* values ≤ 0.05 were considered significant. All analyses were performed in Graphpad Prism v 8.0 (GraphPad Software. Inc.).

For cytokine heatmaps each protein/cytokine was normalized by subtracting the mean value and dividing by standard deviation of the logarithmic transformed values. The normalized values for the cytokines for each subject were shown in the heatmap. The dendrograms and ordering of subjects and cytokines were performed by hierarchical clustering using Ward’s method. Distances between cytokines were calculated by 1 – r, where r is the Pearson correlation coefficient and distances between subjects by Euclidian distance. The R function “agnes” in the “cluster” package was used for clustering.

## Results

### Patient Characteristics

Our study population included 10 patients with MM (4 female and 6 male; mean age, 60.3 years; age range: 39–70), who had undergone HDT treatment within 1–6 months. **Table 1** shows patient characteristics. Nine patients were included after first-line induction therapy with HDT. Induction therapy comprised standard of care therapy of cyclophosphamide, bortezomib, and dexamethasone (Cy-Vel-Dex) for all but one patient who instead received bortezomib, thalidomide, and dexamethasone (VTD, patient 4) due to renal insufficiency at diagnosis. Patient 9 was included after Cy-Vel-Dex and HDT, following relapse occurring 18 years after primary double transplantation. Five patients lacked cytogenetic data from diagnosis. FISH was performed on BM samples at inclusion, but the low tumor burden post-HDT prohibited cytogenetic analysis. All included patients received at least 6 vaccines and are included in the data set.

**Table 1:** Patient characteristics.

	Age	Sex	ECOG PS	Comorbidity	Type of paraprotein	LDH at diagnosis	Cytogenetics at diagnosis	ISS/R-ISS at diagnosis	Time from HDT to start of vaccination (days)
Patient 1	70	F	0	Hypertension, Cholecystectomy	Lambda	262	Not enough material	2/2	188
Patient 2	69	M	0	Hypertension, Hypercholesterolemia, CABG, BCC	Kappa	188	amp1q(80%), t(11:14)(100%) = High risk	1/1	70
Patient 3	58	F	1	Hypertension, multinodular goiter	Lambda	246	Normal FISH = Standard risk	3/3	131
Patient 4	58	M	1	None	IgG kappa	172	t(11:14)(91%) = Standard risk	3/2	43
Patient 5	60	F	1	Inguinal hernea	Lambda	141	t(11:14)(96%) = Standard risk	3/3	61
Patient 6	59	M	1	None	IgG kappa	220	del(13q14.3)(96%) del1p(97%) = High risk	1/2	82
Patient 7	60	F	1	Hypertension, Spinal stenosis operata	IgG kappa	260	Not enough material	3/2	41
Patient 8	61	M	1	None	Biclonal IgG kappa IgA kappa	142	Not enough material	2/2	50
Patient 9	69	M	1	None	IgG kappa	175	Not done	Missing	28
Patient 10	39	M	1	None	IgG kappa	148	Not possible	2/2	83

Patient 9 had previously been transplanted, and was enrolled after a HDT treating first relapse.

HDT, high dose chemotherapy with autologous stem cell transplant; ISS, International Staging System; LDH, lactate dehydrogenase.

### Adverse Events

Infections are common in patients with MM, and the infection rate is further increased for at least one1 year post-HDT. The rate of infections and other adverse events was as expected for the population (**Table 2**). Adverse events considered potentially related to vaccination were most commonly injection site reactions and were all transient of nature (**Table 2**). No adverse events above grade 2 were deemed related to the vaccine. No autoimmune adverse events were observed.

**Table 2:** AEs total adverse events during vaccinations.

Relation to therapy*	Adverse event	No. of patients	Grade 1	Grade 2	Grade 3
Cy-Vel-Dex induction, HDT, myeloma or unrelated	Cold	6	6		
	Respiratory tract infection	3	1		2
	Influenza	2		1	1
	Urinary tract infection	2		2	
	Abscessus	1		1	
	Conjunctivitis	1		1	
	Fungal skin infection	1		1	
	Flu-like viral infection	1		1	
	Gastroenteritis	1		1	
	Herpes reactivation	1		1	
	Sinusitis	1		1	
	Tonsillitis	1			3
	Cough	2	2		
	Diarrhoea	2	2		
	Basal cell carcinoma	1			1
	Constipation	1	1		
	Creatinin increase	1	1		
	Hernia, inguinal	1			1
	Nausea	1		1	
	Palpitations	1		1	
	Sore throat	1	1		
	Tendernes of jaw	1	1		
	Artroscopic miniscus manipulation	1			1
					
PD-L1 vaccine (IO103)	Injection site reaction	9	6	3	
	Pruritus	3	2	1	
	Myalgia	3	1	2	
	Artralgia	2		2	
	Sore nipple	2	2		
	Dry skin	1	1		
	Lymphopenia	1	1		
	Cough	1	1		
	Dermatitis	1		1	
	Rash	2	1	1	
	Swelling of bursa olecrani	1	1		

*Investigator deemed whether adverse events were related or possibly related to the experimental treatment or to other causes. Injection site reactions included local erythema, oedema, and pruritus. Non-tender subcutaneous lumps up to 1 cm in diameter could linger up to months, as is seen commonly with the deposition of the adjuvant Montanide.

Cy-Vel-Dex, cyclophosphamide-bortezomib-dexamethazone; HDT, high-dose chemotherapy with autologous stem cell transplantation.

### Immune Responses in Blood

**Figure 1:**
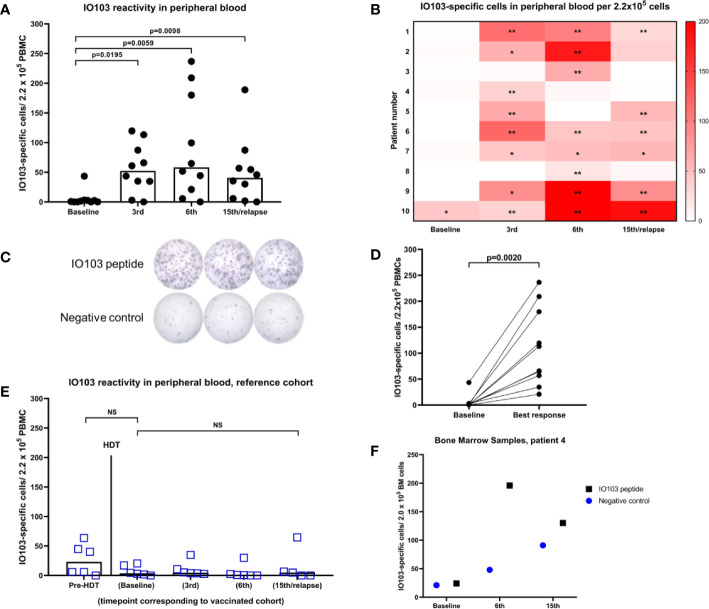
IFNγ-ELISPOT immune responses against IO103. **(A)** responses in PBMCs in vaccinated patients (bars represent median); **(B)** Heatmap of responses in PBMCs per vaccinated per time point. (*DFRx1; **DFRx2); **(C)** representative example of ELISPOT-wells with response; **(D)** best response in PBMCs in vaccinated patients; **(E)** responses in unvaccinated reference cohort including time point before HDT (All p-values: Wilcoxon matched-pairs signed rank test); **(F)** IFNγ-ELISPOT responses against IO103 in bone marrow samples from patient 4.

PBMCs from blood samples were assessed using indirect ELISPOT assays against IO103. No or little response to the vaccine occurred at baseline, while all patients exhibited a response to IO103 during the vaccination course (**Figures 1A–D**). To assess whether immune responses to IO103 would normally occur post-HDT, we ran IO103 ELISPOT on our reference cohort at similar time points as in the vaccinated patients. Consistent with our previous data, we observed spontaneous immune responses to IO103 before HDT. These responses were not observed post-HDT in the unvaccinated reference cohort (**Figure 1E**), likely due to the strong lympho-depleting chemotherapy.

### Immune Responses in the Skin

**Figure 2:**
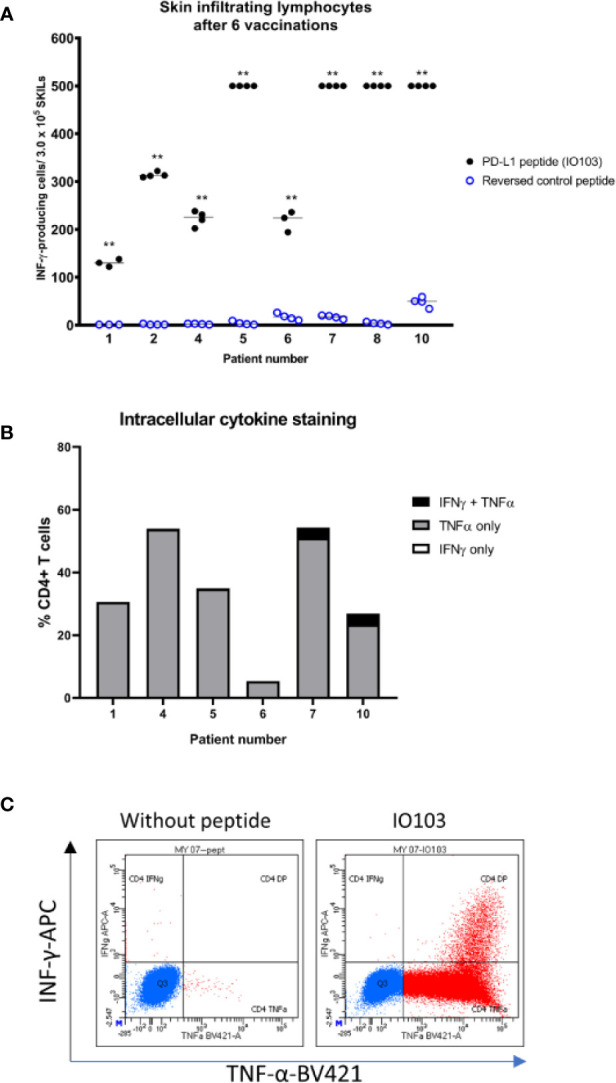
Immune responses to the vaccine in the skin. **(A)** IFNγ-ELISPOT responses against the PD-L1 peptide IO103 of skin infiltrating lymphocytes after delayed type hypersensitivity reaction performed after 6 vaccines. 300,000 cells per well. Samples were run in triplicate or quadruplicate. **DFRx2; **(B)** intracellular cytokine staining (ICS) of SKILs. Almost all cells were CD4+ (not shown). Two out of six evaluable patients had TNFα- and INFγ-double positive SKILs; **(C)** example of a double positive ICS (patient 7).

Nine patients consented to DTH testing before the seventh vaccine. The cells from one patient (patient 3) were accidentally infected in culture. All eight evaluable patients had a positive skin induration of more than double the diameter of the control injection. SKILs could be grown from skin biopsies of all patients, and all were strongly reactive to the vaccine as evaluated by IFNγ-ELISPOT (**Figure 2A**). IL2-expanded SKILs were mainly CD4^+^ T cells (data not shown). Intracellular cytokine staining after stimulation with IO103 revealed that these CD4^+^ SKILs secreted tumor necrosis factor alfa (TNF-α) and a minor fraction also secreted IFN-γ (**Figures 2B, C**).

### Immune Responses in the Bone Marrow

ELISPOT assays on cryopreserved BM samples had a very high background signal due to ex vivo cell death. Reduced viability of BM mononuclear cells is well known (35). The fact that these BM samples were taken in the recovery from HDT, may have decreased the viability further. The low viability of the immune cells in the BM samples did not permit in depth immune monitoring with functional living cells. Nonetheless, singlet samples were run with only modest background in one of three tested patients, and in samples from this patient, a strong immune response to IO103 was seen in the BM (**Figure 1F**).

### Lymphocytes

**Figure 3:**
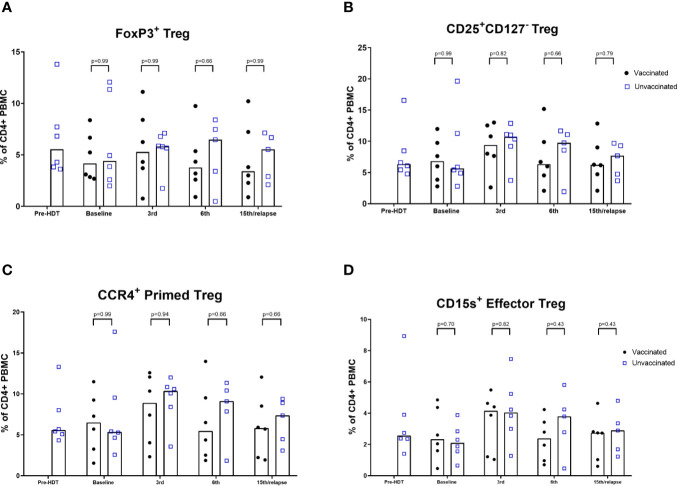
Flow cytometric analyses of levels of Tregs. **(A)** FoxP3+ Treg. **(B)** CD25highCD127neg Treg; **(C)** CCR4 + Primed Tregs; **(D)** CD15s Effector Tregs. % of CD4+ PBMCs. Bars represent median (Mann-Whitney).

An exploratory analysis of lymphocyte phenotypes was performed comparing samples from the vaccinated patients to samples from the unvaccinated reference cohort. Vaccinated patients 1, 3, 6, and 10 were not included in these comparisons, since these patients did not have synchronous reference cohort samples for comparison. An inversion of the CD4/CD8 ratio following HDT was seen in the unvaccinated cohort, and the vaccinated patients had a similar ratio at baseline (**Supplementary Figures 1A, B**). A significantly lower level of CD4 cells in vaccinated patients after 15^th^ vaccination or relapse was seen. Gating of differentiation stages did not reveal significant differences and exhibited substantial interindividual variance. Naïve cell repopulation post-HDT did not significantly differ in samples from vaccinated patients compared to the reference population (**Supplementary Figures 2** and **3**). The level of Tregs was not found to be significantly different compared to the reference population (**Figures 3A–D**). The FoxP3^+^ Treg levels of all vaccinated patients are shown in **Supplementary Figure 12**. Levels of DCs in peripheral blood samples did not show significant differences compared to the reference population (**Supplementary Figure 13**).

### Clinical Course

Before vaccinations, four patients were in complete response (CR) or better, and five had very good partial response (VGPR). At the data cut-off (May 14, 2020; mean follow-up, 36.5 months), 2/10 patients had not started their next treatment, and 8/10 were still alive (**Supplementary Figure 4**). The relapse rate was as expected for the population.

**Figure 4:**
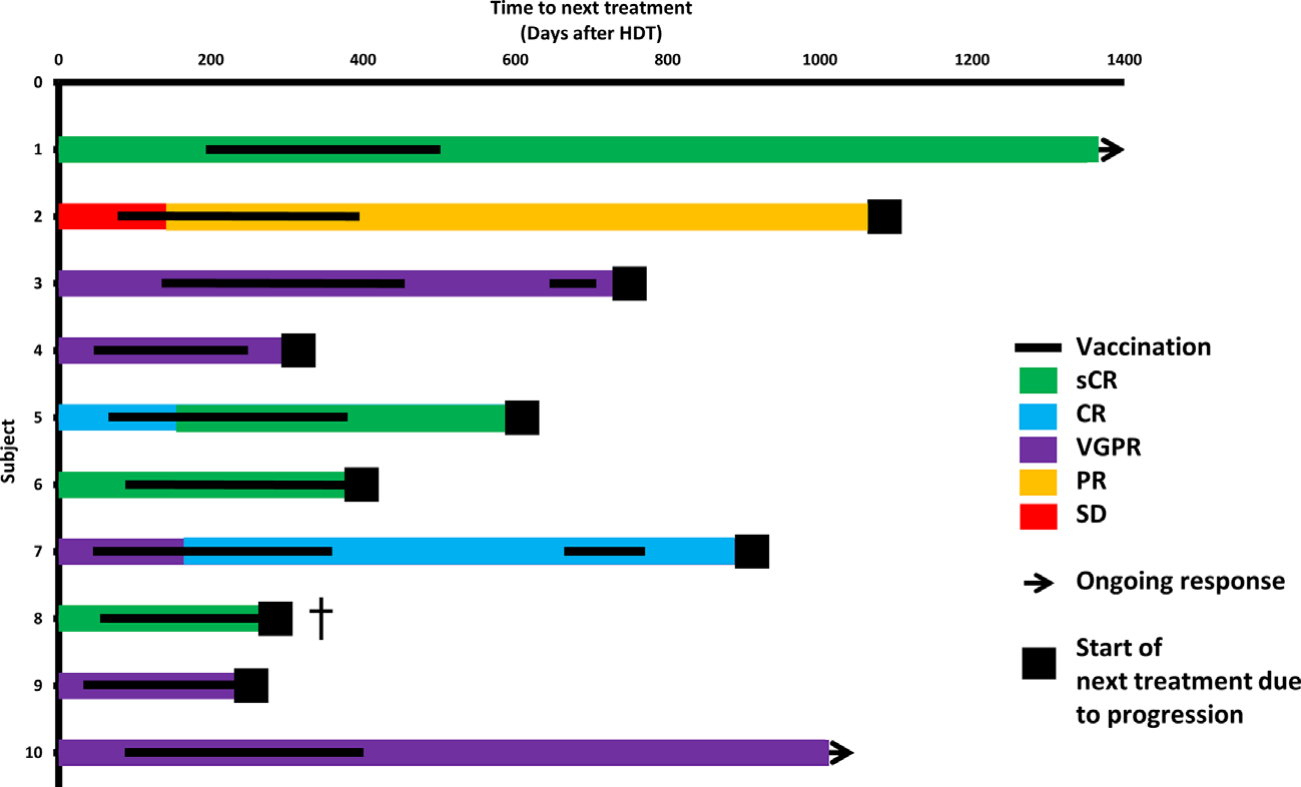
Clinical course. Swimmer’s plot. Colors of bars symbolize depth of response at start of vaccinations, after HDT and during the vaccination course. ^†^patient 8 had a rapid relapse after having received 11 vaccinations and died shortly thereafter despite initiation of daratumumab-lenalidomide-dexamethasone.

Three patients exhibited improved depth of response during vaccination therapy: patient 2 at day 145 post-ASCT (after 5 vaccines), patient 5 at day 161 (6 vaccines), and patient 7 at day 168 (7 vaccines) (**Figure 4**).

Patients 3 and 7 showed declining levels of M component/involved light chain during vaccinations; however, both exhibited slow biochemical relapse after completing vaccinations. As a study amendment, these two patients were revaccinated to test if a stabilization or decline could be induced. In patient 3, revaccination did not decrease the tumor marker slope (**Supplementary Figure 7**). In patient 7, the M-component slope decreased briefly along with revaccinations (**Supplementary Figure 8**).

Patient 8 exhibited an early rapid clinical relapse, which was fatal despite initiation of therapy with daratumumab, lenalidomide, and dexamethasone. At this time, the patient had received 11 vaccines. We explored possible correlations between immune data and clinical course. Patients who did and did not relapse during the vaccination course did not differ in ELISPOT responses to IO103 in blood samples (**Supplementary Figure 5**). However, the two earliest relapsing patients (patients 8 and 9) showed baseline cytokine profiles that grouped apart from the remaining vaccinated patients in unsupervised analysis (**Supplementary Figures 6A, B**). Furthermore, two patients (patients 8 and 4) with early relapses had the lowest ELISPOT responses to IO103 in PBMCs (**Figure 1C**), and the highest Treg levels in PBMCs even at baseline (**Supplementary Figure 11**).

During the vaccination course, patient 2 exhibited significant regression of concurrent basal cell carcinoma (BCC), and patient 6 exhibited spontaneous BCC clearance that was macroscopically complete, such that planned surgery was cancelled. In patient 6, BCC recurred, coinciding with biochemical relapse of MM.

## Discussion

The vaccine was generally well tolerated, and the frequency of adverse events was as expected in the patient population. Adverse events that were related or possibly related to vaccination with IO103 were mild (CTCAE grade 1–2) and reversible, and most frequently injection site reactions.

PD-L1 is expressed on cancer cells and non-cancerous cells in the tumor microenvironment, on normal antigen-presenting cells and placental cells, and frequently on cells in an inflammatory microenvironment, since its expression is primarily regulated by interferons (36). We recently demonstrated that inflammation alone induces PD-L1–specific T cells (37). The fact that PD-L1–specific T cells are so easily and rapidly activated, but do not lead to outright autoimmune reactions, implies that these cells are strictly regulated. Mouse studies of vaccination with murine PD-L1 peptides confirm the absence of signs of autoimmune events (in preparation). In this first-in-human study, we observed immune reactions to the vaccine in blood samples and skin tests from all patients. It is intriguing that stimulation of these self-reactive T cells strongly induced immune responses in all patients, with low toxicity.

In the months following lymphodepleting chemotherapy in HDT, CD8^+^ T-cell repopulation is aided by homeostatic peripheral expansion more than the CD4^+^ compartment, yielding a relatively low Treg/CD8^+^ ratio, which gradually normalizes as levels return to baseline (25). An inverted ratio of CD4^+^/CD8^+^ T cells was found in both vaccinated patients and the unvaccinated reference cohort. In preclinical studies, activation of PD-L1–specific T-cells reduced Treg levels in autologous PBMCs (37). Vaccination against an indoleamine 2,3-dioxygenase (IDO) peptide in patients with lung cancer similarly induced a significant decrease in Tregs (38). IDO is another self-antigen expressed in regulatory immune cells, which is recognized by pro-inflammatory T cells (39, 40). In the present study, although the proportion of CD4^+^ T cells was lower in vaccinated patients at the last time point, the levels of Tregs was not significantly lower among vaccinated patients at any time point. This study setting might not permit the assessment of the vaccine’s effect on Treg levels, due to the recently been exposure to lymphodepleting chemotherapy.

Antigen presenting cells at the vaccination site may be PD-L1 positive. Thus, vaccination induced elimination of these cells could impact antigen presentation at the vaccination site. However, analysis of DC infiltration at the vaccination site was not been performed in this study but will be part of future investigations. Importantly, no signs of a major effect of vaccinations were seen on the frequencies of DC in peripheral blood.

One limitation of this study was our inability to thoroughly investigate immune responses in BM samples. Since MM is a disease of BM-residing cells, one would hope to see an immune response in the BM. However, the viability of BM samples precluded other functional assays of BM immune cells, and assessment of immune responses at the tumor site. These limitations led us to instead analyze the impact of vaccinations on T-cell-receptor (TCR) diversity in SKILs and BM samples, including PD-L1–specific TCRs. These studies are currently on-going.

The current study was valuable for examining the vaccine’s safety. The minimal tumor burden post-HDT allowed us to vaccinate many times, over a prolonged period. Phase one studies are often performed in the relapsed or refractory setting, which would likely not leave sufficient time to test the vaccine’s safety before disease progression. One downside of the post-HDT setting is that clinical efficacy was difficult to investigate. Nine patients were vaccinated post-HDT as part of primary treatment of newly diagnosed MM. In this patient population, median progression-free survival without maintenance treatment is 32 months, and 20% of patients will be in long-term remission (6 years post-HDT) (41). With a median follow-up of 29 months, the PFS data are not yet mature. In a Mayo Clinic study, response improvements after day +100 occurred in up to 39% of cases (42). However, among patients treated in Barcelona and Salamanca, only 1/74 patients had an upgraded response after day 100 without maintenance or consolidation (43). Response improvements beyond day 100 rarely occur at our institution (personal communication), and late improvements have been used as an argument regarding response to immunotherapy after HDT (12). The three late response improvements (over 100 days post-HDT), and one case of short-term stabilization coinciding with revaccination, may indicate the vaccine’s efficacy.

The incidental findings of spontaneous BCC regression in patients 2 and 6 are interesting. After the initial spontaneous BCC clearance, patient 6 experienced clinical relapse of BCC coinciding with biochemical relapse of MM. This suggests that the vaccine induced a response toward the BCC, which lasted for months, and then experienced a loss of immune control affecting both malignancies once the vaccination was no longer effective. This cannot be tested with current methods but has prompted the initiation of a phase IIa study of vaccination with IO103 in BCC (NCT03714529).

Based on the present promising safety and immune data, several clinical trials have been initiated. It is hypothesized that therapeutic cancer vaccines, without chemotherapy or ICB, are likely to be most effective if administered in early disease stages (44, 45). Thus, a trial of IO103 in high-risk smoldering myeloma has been initiated (NCT03850522). Additionally, we are testing this vaccine combined with other peptide vaccines against PD-L2, IDO, and arginase, and with immune checkpoint-blocking antibodies (NCT03381768, NCT03939234, and NCT04051307).

In conclusion, the vaccination against PD-L1 was easily administered and was associated with few and low-grade and reversible side effects. Furthermore, the vaccine induced strong immune responses in all patients.

## Data Availability Statement

The raw data supporting the conclusions of this article will be made available by the authors, without undue reservation.

## Ethics Statement

The studies involving human participants were reviewed and approved by De Videnskabsetiske Komiteer i Region Hovedstaden. The patients/participants provided their written informed consent to participate in this study.

## Author Contributions

NJ designed the study, obtained institutional approval, performed the research, analyzed data, and wrote the manuscript. UK, JG, CH, TA, and TD recruited and treated the patients and critically revised the manuscript. SA, MB, EM, and ÖM performed laboratory research and critically revised the manuscript. LO, TK, and MH assisted in the data analysis and critically revised the manuscript. IS, LK, and MA conceptualized the study, designed the study, supervised the project, and critically revised the manuscript. All authors contributed to the article and approved the submitted version.

## Funding

The work was funded by The Danish Cancer Society, The Copenhagen University Hospital Herlev and Gentofte, and through a research funding agreement between IO Biotech ApS and National Center for Cancer Immune Therapy (CCIT-DK).

## Conflict of Interest

NJ: Research position is funded by The Danish Cancer Society; Copenhagen University Hospital, Herlev and Gentofte; and through a research funding agreement between IO Biotech ApS and National Center for Cancer Immune Therapy (CCIT-DK). MA: Author of a filed patent application based on the use of the PD-L1 peptide IO103 for vaccination. The patent rights have been transferred to Copenhagen University Hospital, Herlev, and Gentofte/The Capital Region of Denmark, in accordance with the Danish Law of Public Inventions at Public Research Institutions. Additionally, the author is a shareholder and board member of the company IO Biotech ApS, which has the purpose of developing commercial PD-L1 vaccines for cancer treatment. IS: Co-founder and shareholder in IOBiotech Aps.

The remaining authors declare that the research was conducted in the absence of any commercial or financial relationships that could be construed as a potential conflict of interest.

## References

[1] Bedognetti D Ceccarelli M Galluzzi L Lu R Palucka K Samayoa J Toward a comprehensive view of cancer immune responsiveness: A synopsis from the SITC workshop. J Immunother Cancer (2019) 7:1–23. 10.1186/s40425-019-0602-4 31113486 PMC6529999

[2] Naidoo J Page DB Li BT Connell LC Schindler K Lacouture ME Toxicities of the Anti-PD-1 and Anti-PD-L1 Immune Checkpoint Antibodies. Ann Oncol (2015) 26:2375–91. 10.1093/annonc/mdv383 PMC6267867 26371282

[3] Munir S Andersen GH Met Ö Donia M Frøsig TM Larsen SK HLA-restricted CTL that are specific for the immune checkpoint ligand PD-L1 occur with high frequency in cancer patients. Cancer Res (2013) 73:1764–76. 10.1158/0008-5472.CAN-12-3507 23328583

[4] Munir S Andersen GH Woetmann A Ødum N Becker JC Andersen MH.Cutaneous T cell lymphoma cells are targets for immune checkpoint ligand PD-L1-specific, cytotoxic T cells. Leukemia (2013) 27:2251–2253. 10.1038/leu.2013.118 23660624

[5] Ahmad SM Larsen SK Svane IM Andersen MH Harnessing PD-L1-specific cytotoxic T cells for anti-leukemia immunotherapy to defeat mechanisms of immune escape mediated by the PD-1 pathway. Leukemia (2013) 28:236–8. 10.1038/leu.2013.261 24091833

[6] Holmström MO Riley CH Skov V Svane IM Hasselbalch HC Andersen MH.Spontaneous T-cell responses against the immune check point programmed-death-ligand 1 (PD-L1) in patients with chronic myeloproliferative neoplasms correlate with disease stage and clinical response. Oncoimmunology (2018) 7:1–6. 10.1080/2162402X.2018.1433521 PMC5980374 29872567

[7] Munir S Andersen GH Svane IM Andersen MH The immune checkpoint regulator PD-L1 is a specific target for naturally occurring CD4(+) T cells. Oncoimmunology (2013) 2:e23991. 10.4161/onci.23991 23734334 PMC3654604

[8] Munir Ahmad S Martinenaite E Hansen M Junker N Borch TH Met Ö PD-L1 peptide co-stimulation increases immunogenicity of a dendritic cell-based cancer vaccine. Oncoimmunology (2016) 5:e1202391. 10.1080/2162402X.2016.1202391 27622072 PMC5007957

[9] Benson DM Bakan CE Mishra A Hofmeister CC Efebera Y Becknell B The PD-1 / PD-L1 axis modulates the natural killer cell versus multiple myeloma effect : a therapeutic target for CT-011, a novel monoclonal anti – PD-1 antibody. Blood (2010) 116:2286–94. 10.1182/blood-2010-02-271874 20460501 PMC3490105

[10] Liu J Hamrouni A Wolowiec D Coiteux V Kuliczkowski K Hetuin D Plasma cells from multiple myeloma patients express B7-H1 (PD-L1) and increase expression after stimulation with IFN-γ and TLR ligands via a MyD88-, TRAF6-, and MEK-dependent pathway. Blood (2007) 110:296–304. 10.1182/blood-2006-10-051482 17363736

[11] Hallett WHD Jing W Drobyski WR Johnson BD Immunosuppressive Effects of Multiple Myeloma Are Overcome by PD-L1 Blockade. Biol Blood Marrow Transplant (2011) 17:1133–45. 10.1016/j.bbmt.2011.03.011 21536144

[12] Rosenblatt J Avivi I Vasir B Uhl L Munshi NC Katz T Vaccination with dendritic cell/tumor fusions following autologous stem cell transplant induces immunologic and clinical responses in multiple myeloma patients. Clin Cancer Res (2013) 19:3640–8. 10.1158/1078-0432.CCR-13-0282 23685836 PMC3755905

[13] Ray A Das DS Song Y Richardson P Munshi NC Chauhan D Targeting PD1–PDL1 immune checkpoint in plasmacytoid dendritic cell interactions with T cells, natural killer cells and multiple myeloma cells. Leukemia (2015) 29:1441–4. 10.1038/leu.2015.11 25634684 PMC5703039

[14] Sponaas AM Moharrami NN Feyzi E Standal T Rustad EH Waage A PDL1 expression on plasma and dendritic cells in myeloma bone marrow suggests benefit of targeted anti PD1-PDL1 therapy. PloS One (2015) 10:e0139867. 10.1371/journal.pone.0139867 26444869 PMC4596870

[15] An G Acharya C Feng X Wen K Zhong M Zhang L Osteoclasts promote immune suppressive microenvironment in multiple myeloma: therapeutic implication. Blood (2016) 128:1590–1603. 10.1182/blood-2016-03-707547 27418644 PMC5034739

[16] Paiva B Azpilikueta A Puig N Ocio EM Sharma R Oyajobi BO PD-L1/PD-1 presence in the tumor microenvironment and activity of PD-1 blockade in multiple myeloma. Leukemia (2015) 29:2110–3. 10.1038/leu.2015.79 25778100

[17] Ishibashi M Tamura H Sunakawa M Kondo-Onodera A Okuyama N Hamada Y Myeloma Drug Resistance Induced by Binding of Myeloma B7-H1 (PD-L1) to PD-1. Cancer Immunol Res (2016) 4:779–88. 10.1158/2326-6066.CIR-15-0296 27440711

[18] Crescenzi A Annibali O Bianchi A Pagano A Donati M Grifoni A PD-1/PD-L1 expression in extra-medullary lesions of multiple myeloma. Leuk Res (2016) 49:98–101. 10.1016/j.leukres.2016.09.008 27619200

[19] Gasmi B Smith E Dogan A Hsu M Devlin S Pichardo J Presence of PD-1 Expressing T Cells Predicts for Inferior Overall Survival in Newly Diagnosed Multiple Myeloma. ASH 2015 (abstract 1785) (2015). Available at: https://ash.confex.com/ash/2015/webprogram/Paper84149.html (Accessed 10th December 2015).

[20] Tamura H Ishibashi M Yamashita T Tanosaki S Okuyama N Kondo A Marrow stromal cells induce B7-H1 expression on myeloma cells, generating aggressive characteristics in multiple myeloma. Leukemia (2013) 27:464–472. 10.1038/leu.2012.213 22828443

[21] Lesokhin AM Ansell SM Armand P Scott EC Halwani A Gutierrez M Nivolumab in Patients with Relapsed or Refractory Lymphoid Malignancy: Preliminary Results of a Phase I Study. J Clin Oncol (2016) 34:2698–704. 10.1200/JCO.2015.65.9789 PMC5019749 27269947

[22] Usmani SZ Schjesvold F Oriol A Karlin L Cavo M Rifkin RM Pembrolizumab plus lenalidomide and dexamethasone for patients with treatment-naive multiple myeloma (KEYNOTE-185): a randomised, open-label, phase 3 trial. Lancet Haematol (2019) 6:e448–58. 10.1016/s2352-3026(19)30109-7 31327689

[23] Mateos M-V Blacklock H Schjesvold F Oriol A Simpson D George A Pembrolizumab plus pomalidomide and dexamethasone for patients with relapsed or refractory multiple myeloma (KEYNOTE-183): a randomised, open-label, phase 3 trial. Lancet Haematol (2019) 6:e459–69. 10.1016/s2352-3026(19)30110-3 31327687

[24] van der Burg SH Arens R Ossendorp F van Hall T Melief CJM Vaccines for established cancer: overcoming the challenges posed by immune evasion. Nat Rev Cancer (2016) 16:219–233. 10.1038/nrc.2016.16 26965076

[25] Chung DJ Pronschinske KB Shyer JA Sharma S Leung S Curran SA T-cell Exhaustion in Multiple Myeloma Relapse after Autotransplant: Optimal Timing of Immunotherapy. Cancer Immunol Res (2016) 4:61–71. 10.1158/2326-6066.CIR-15-0055 26464015 PMC4703436

[26] Svane IM Nikolajsen K Johnsen HE Antigen-specific T-cell immunity in multiple myeloma patients is restored following high-dose therapy: implications for timing of vaccination. Scand J Immunol (2007) 66:465–75. 10.1111/j.1365-3083.2007.01993.x 17850592

[27] Williams KM Hakim FT Gress RE T cell immune reconstitution following lymphodepletion. Semin Immunol (2007) 19:318–330. 10.1016/j.smim.2007.10.004 18023361 PMC2180244

[28] Gökbuget N Canaani J Nagler A Bishop M Kröger N Avigan D.Prevention and treatment of relapse after stem cell transplantation with immunotherapy. Bone Marrow Transplant (2018) 53:664–72. 10.1038/s41409-018-0232-3 29795427

[29] Melief CJM van der Burg SH Immunotherapy of established (pre)malignant disease by synthetic long peptide vaccines. Nat Rev Cancer (2008) 8:351–60. 10.1038/nrc2373 18418403

[30] Ascarateil S Puget A Koziol M-E Safety data of Montanide ISA 51 VG and Montanide ISA 720 VG, two adjuvants dedicated to human therapeutic vaccines. J Immunother Cancer (2015) 3:2015. 10.1186/2051-1426-3-S2-P428

[31] Rajkumar SV Harousseau JL Durie B Andersen KC Dimopoulos M Kyle R Consensus recommendations for the uniform reporting of clinical trials: report of the International Myeloma Workshop Consensus Panel 1. Blood (2011) 117:4691–5. 10.1182/blood-2010-10-299487 21292775 PMC3710442

[32] de Vries IJM Bernsen MR Lesterhuis WJ Scharenborg NM Strijk SP Gerritsen MJP Immunomonitoring tumor-specific T cells in delayed-type hypersensitivity skin biopsies after dendritic cell vaccination correlates with clinical outcome. J Clin Oncol (2005) 23:5779–87. 10.1200/JCO.2005.06.478 16110035

[33] McCutcheon M Wehner N Wensky A Kushner M Doan S Hsiao L A sensitive ELISPOT assay to detect low-frequency human T lymphocytes. J Immunol Methods (1997) 210:149–66. 10.1016/S0022-1759(97)00182-8 9520298

[34] Moodie Z Price L Janetzki S Britten CM Response Determination Criteria for ELISPOT: Toward a Standard that Can Be Applied Across Laboratories. Methods Mol Biol (2012) 792:185–96. 10.1007/978-1-61779-325-7_15 21956511

[35] Yang B Parsha K Schaar K Satani N Xi X Aronowski J Cryopreservation of Bone Marrow Mononuclear Cells Alters Their Viability and Subpopulation Composition but Not Their Treatment Effects in a Rodent Stroke Model. Stem Cells Int (2016) 2016:5876836. 10.1155/2016/5876836 27403167 PMC4926012

[36] Garcia-Diaz A Shin DS Moreno BH Saco J Escuin-Ordinas H Rodriguez GA Interferon Receptor Signaling Pathways Regulating PD-L1 and PD-L2 Expression. Cell Rep (2017) 19:1189–201. 10.1016/j.celrep.2017.04.031 28494868 PMC6420824

[37] Munir S Lundsager MT Jørgensen MA Hansen M Petersen TH Bonefeld CM Inflammation induced PD-L1-specific T cells. Cell Stress (2019) 3:319–327. 10.15698/cst2019.10.201 31656949 PMC6789434

[38] Iversen TZ Engell-Noerregaard L Ellebaek E Andersen R Larsen SK Bjoern J Long-lasting disease stabilization in the absence of toxicity in metastatic lung cancer patients vaccinated with an epitope derived from indoleamine 2,3 dioxygenase. Clin Cancer Res (2014) 20:221–32. 10.1158/1078-0432.CCR-13-1560 24218513

[39] Sørensen RB Hadrup SR Svane IM Hjortsø MC Straten PT Andersen MH.Indoleamine 2,3-dioxygenase specific, cytotoxic T cells as immune regulators. Blood (2011) 117:2200–10. 10.1182/blood-2010-06-288498 21079151 PMC3062329

[40] Andersen MH The balance players of the adaptive immune system. Cancer Res (2018) 78:1379–1382. 10.1158/0008-5472.CAN-17-3607 29440147

[41] Danish Myeloma Study Group Danish Myeloma Database. (2017). Available at www.myeloma.dk.

[42] Gonsalves WI Gertz MA Dispenzieri A Lacy MQ Lin Y Singh PP Implications of continued response after autologous stem cell transplantation for multiple myeloma. Blood (2013) 122:1746–49. 10.1182/blood-2013-03-492678 23863899 PMC3765057

[43] Fernández De Larrea C Dávila J Isola I Ocio EM Rosinol L García-Sanz R Absence of spontaneous response improvement beyond day +100 after autologous stem cell transplantation in multiple myeloma. Bone Marrow Transplant (2017) 52:567–9. 10.1038/bmt.2016.299 27869809

[44] Tran T Blanc C Granier C Saldmann A Tanchot C Tartour E.Therapeutic cancer vaccine: building the future from lessons of the past. Semin Immunopathol (2018) 41:69–85. 10.1007/s00281-018-0691-z 29978248

[45] van der Burg SH Correlates of immune and clinical activity of novel cancer vaccines. Semin Immunol (2018) 39:119–36. 10.1016/j.smim.2018.04.001 29709421

