# Supplementary material for: Peptide Vaccination Against PD-L1 With IO103 a Novel Immune Modulatory Vaccine in Multiple Myeloma: A Phase I First-in-Human Trial
Source: Front Immunol. 2020 Nov 9;11:595035. doi: 10.3389/fimmu.2020.595035 (PMC7680803; doi:10.3389/fimmu.2020.595035)
Supplement: Supplementary file 1 [file DataSheet_1.docx]

**Supplementary material**

**Peptide vaccination against PD-L1 with IO103 a novel immune modulatory vaccine in multiple myeloma: a phase I first-in-human trial**

**Summary:**

In this first in human study, 10 patients with multiple myeloma were vaccinated with IO103, a peptide from PD-L1 after high-dose chemotherapy with stem cell support. Primary endpoint was safety.

**Supplementary Table 1
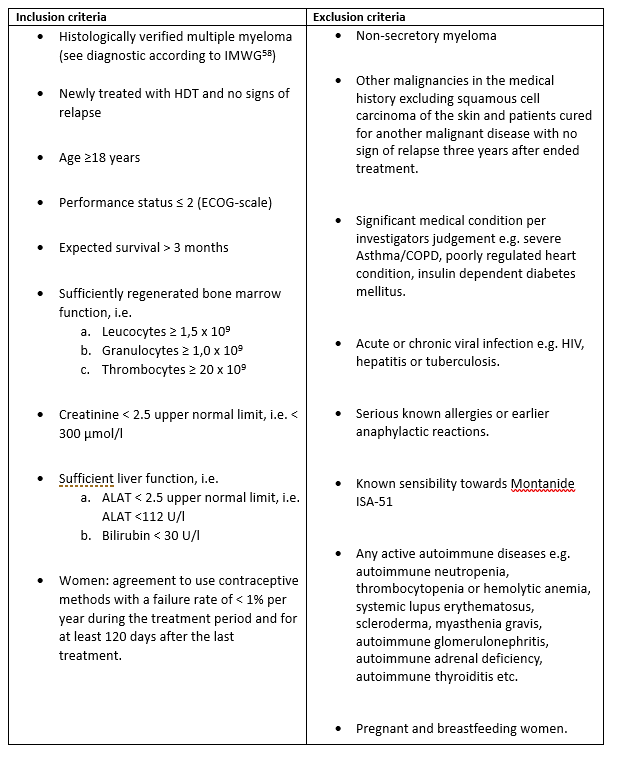

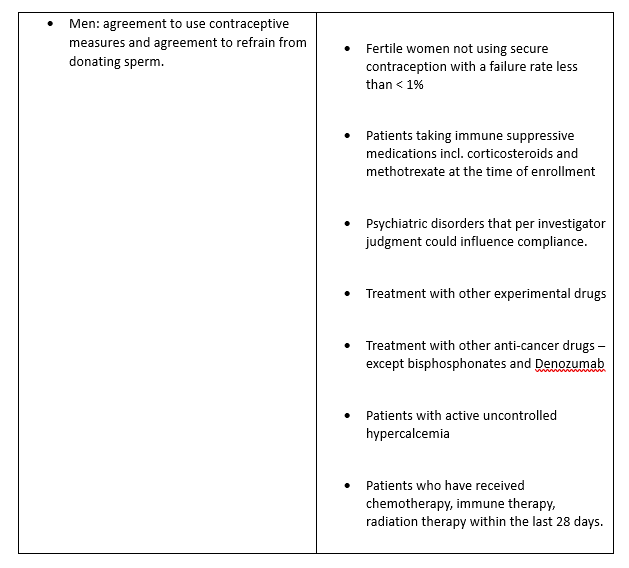
**

**Supplementary Figure 1**

**a b**

**
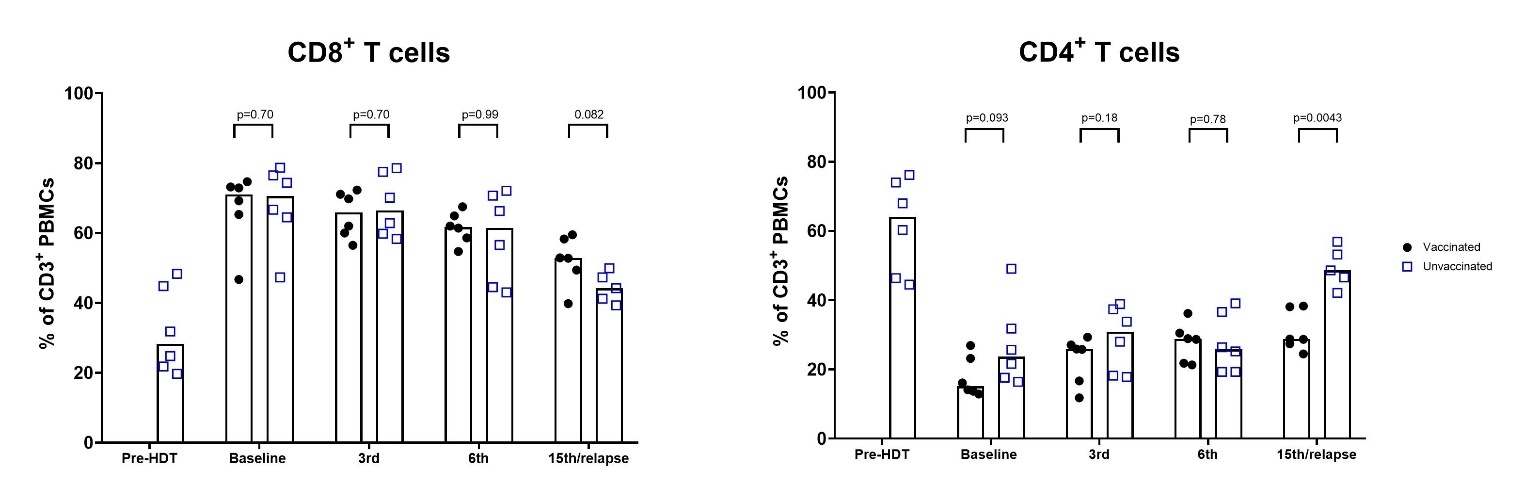
**

**Flowcytometric analysis of frequency of CD8^+^ and CD4^+^ T cells.**

**Supplementary Figure 2**

**
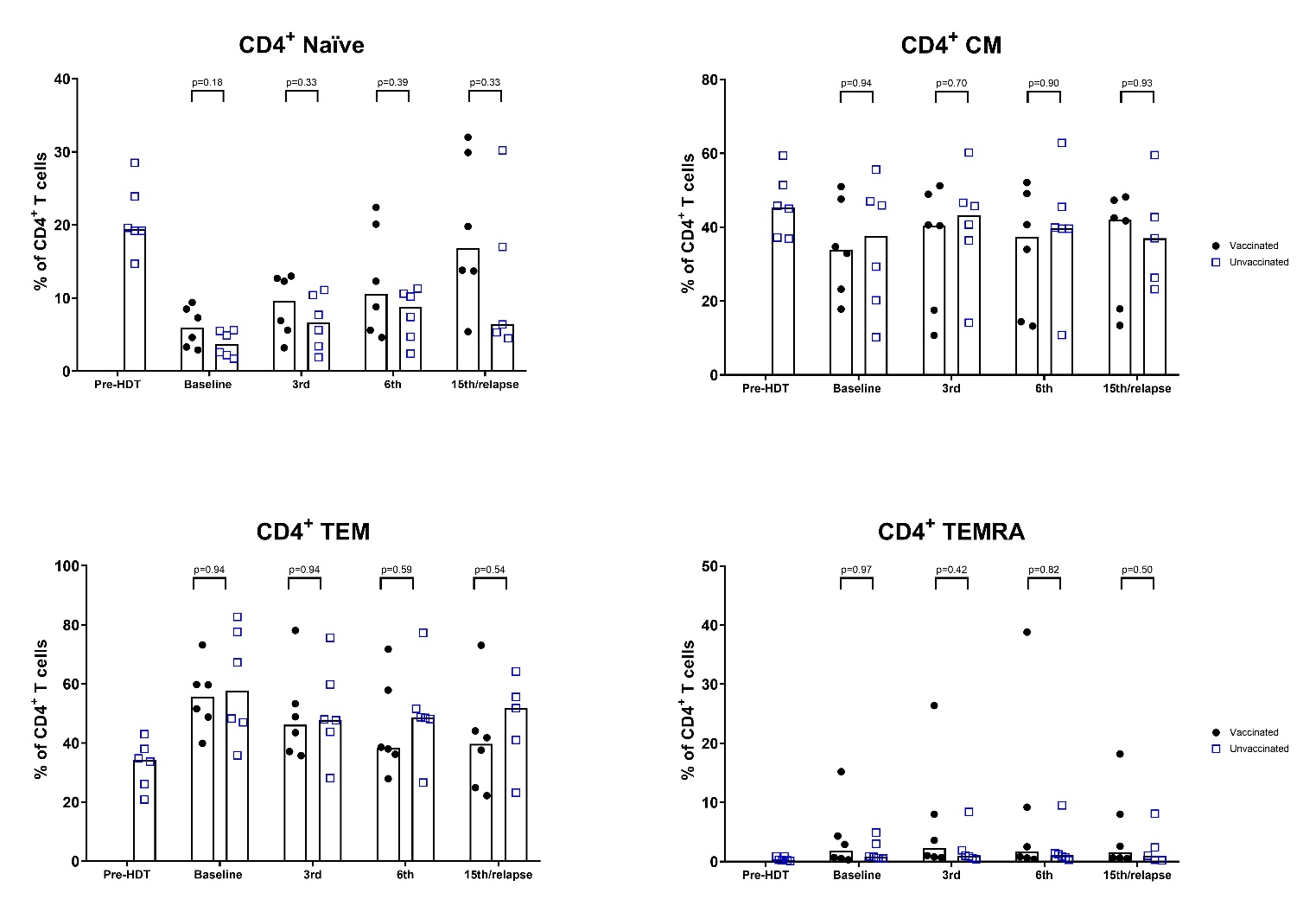
**

**Flowcytometric analysis of differentiation of CD4+ T cells.** CM: Central Memory. TEM: Effector Memory. TEMRA: CD45RA+ Effector Memory

**Supplementary Figure 3**

**
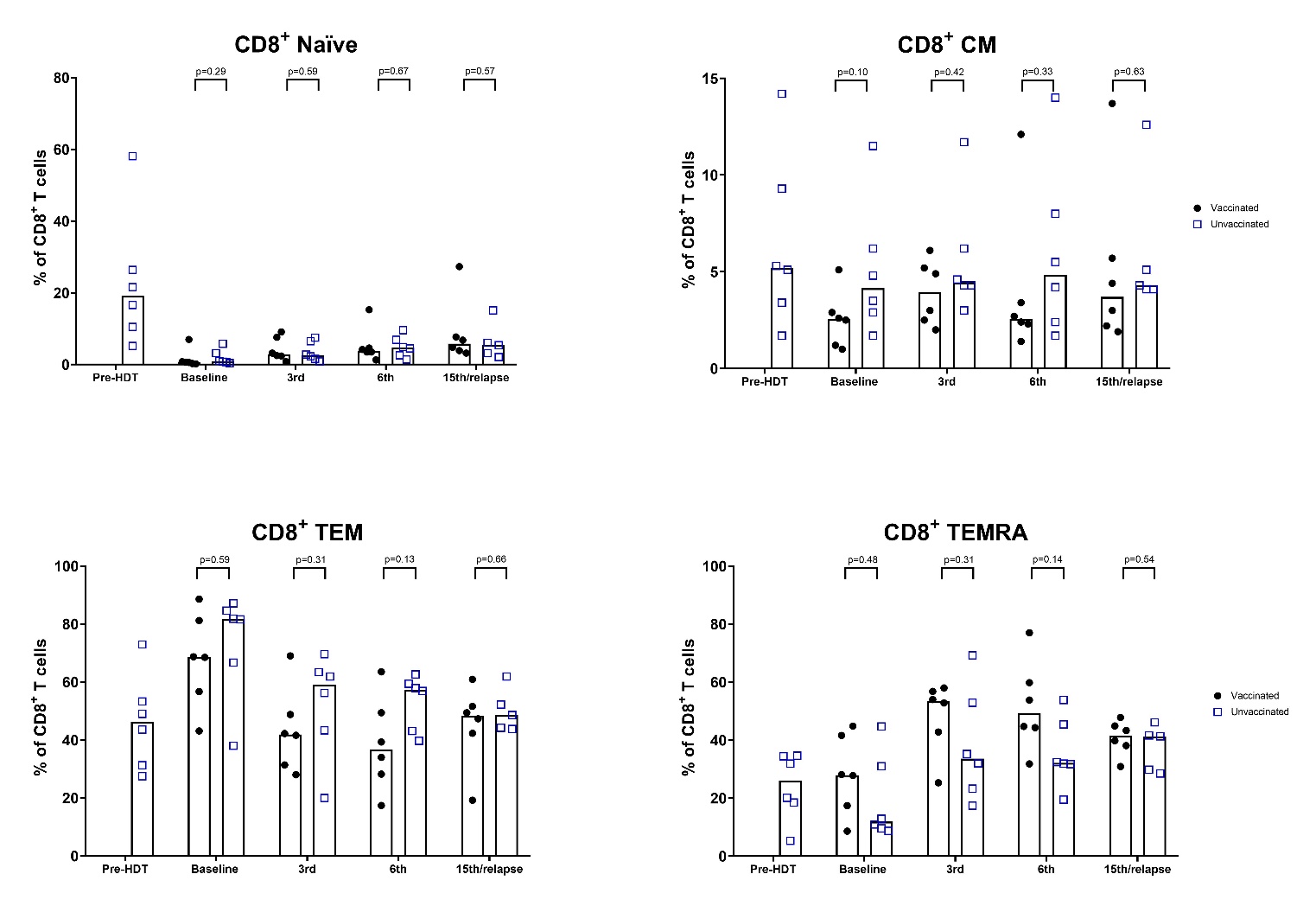
**

**Flowcytometric analysis of differentiation of CD8+ T cells.** CM: Central Memory. TEM: Effector Memory. TEMRA: CD45RA+ Effector Memory

**Supplementary fig 4**

**
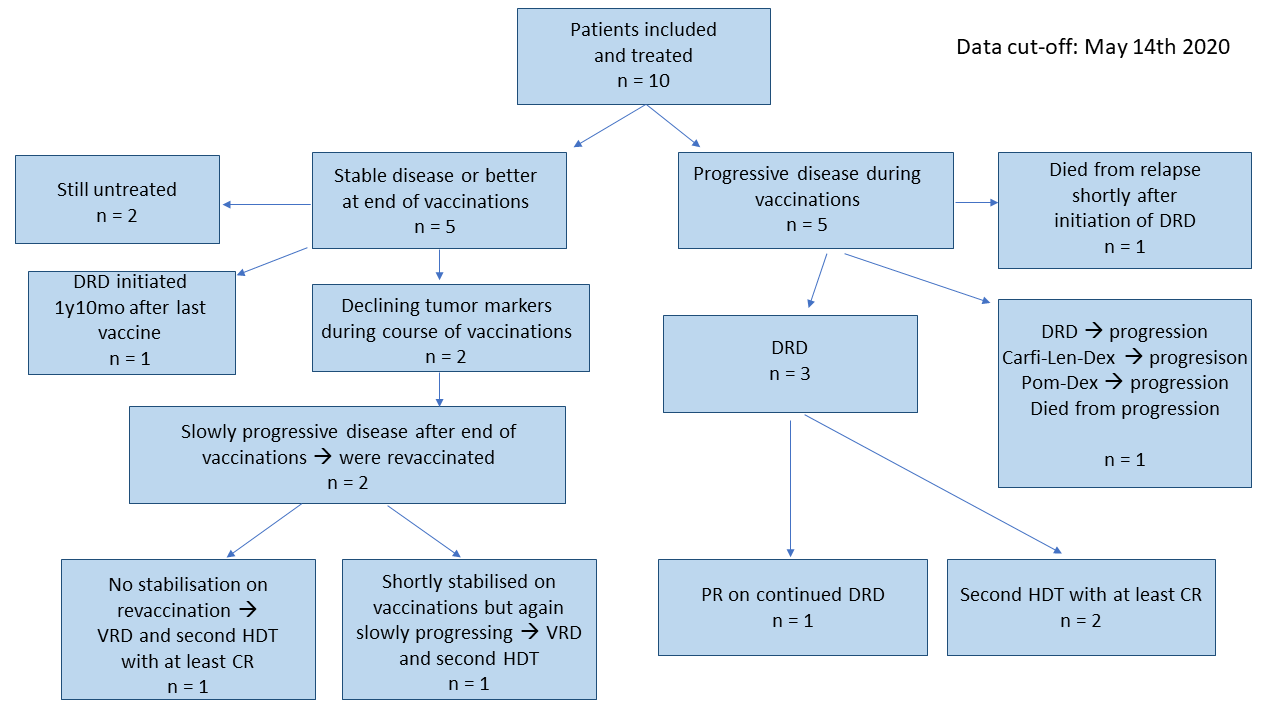
**

**Supplementary Figure 5**

**
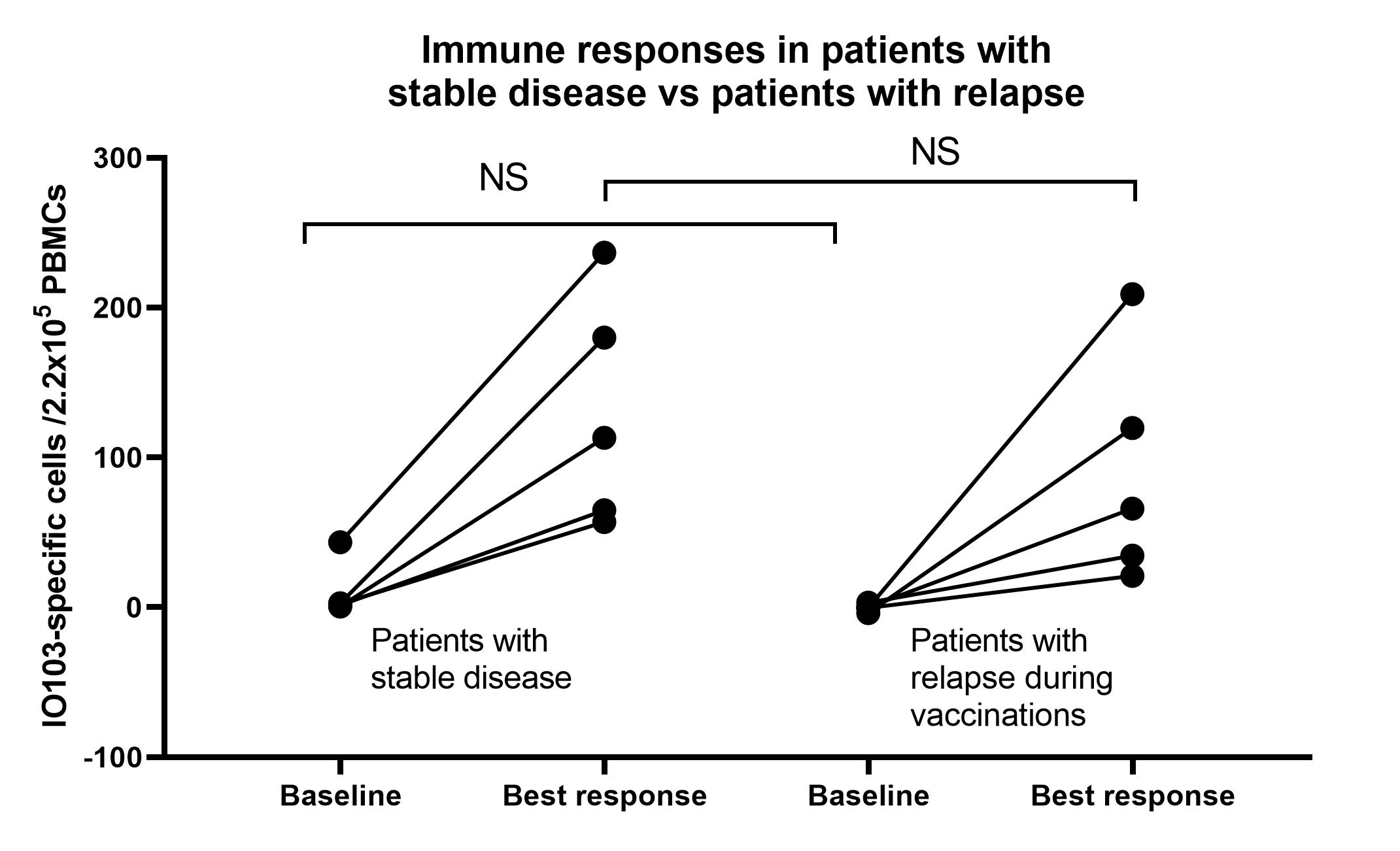
**

**Supplementary Figure 6**

**a**

**
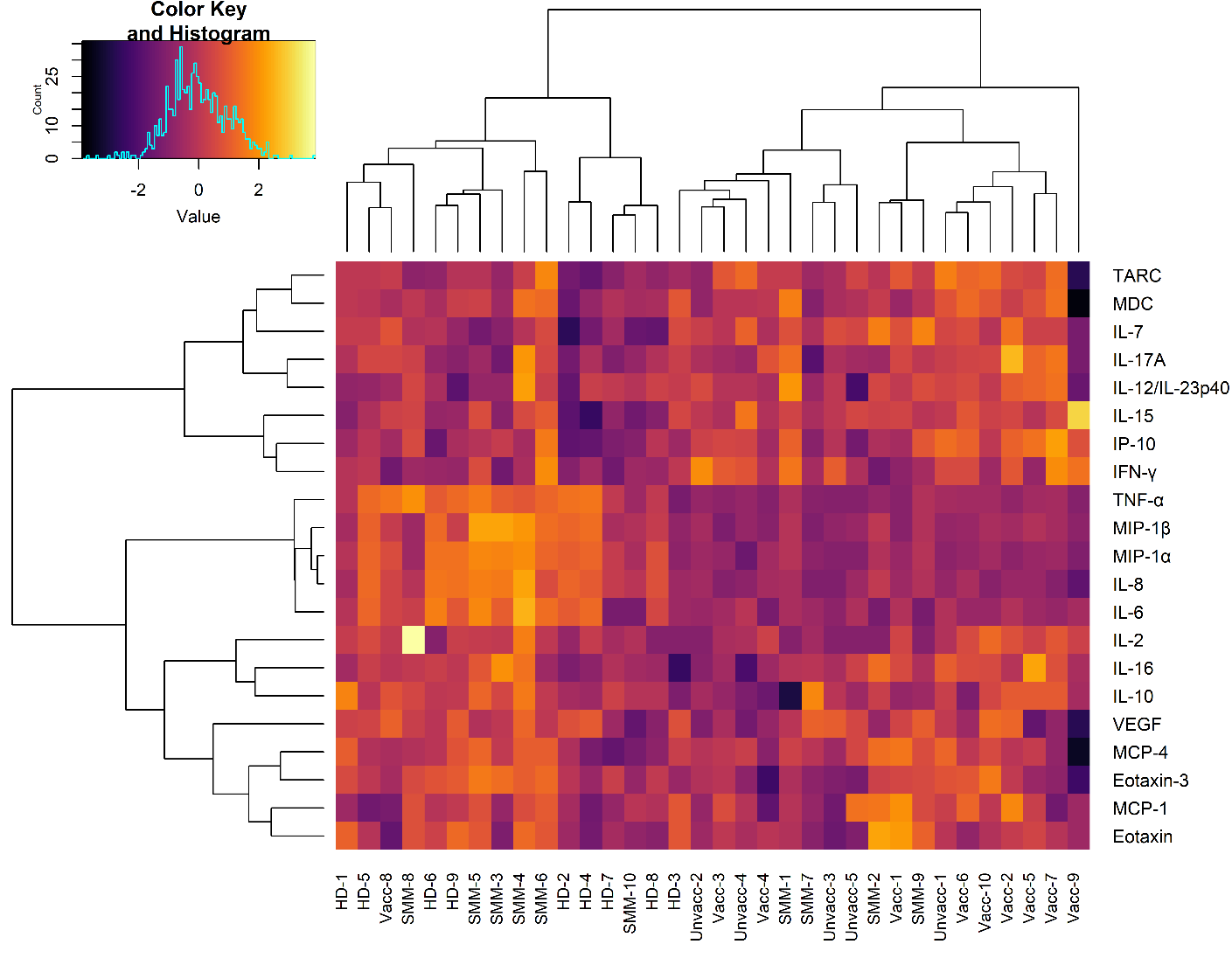
**

**Supplementary figure 6 (cont)**

**b**

**
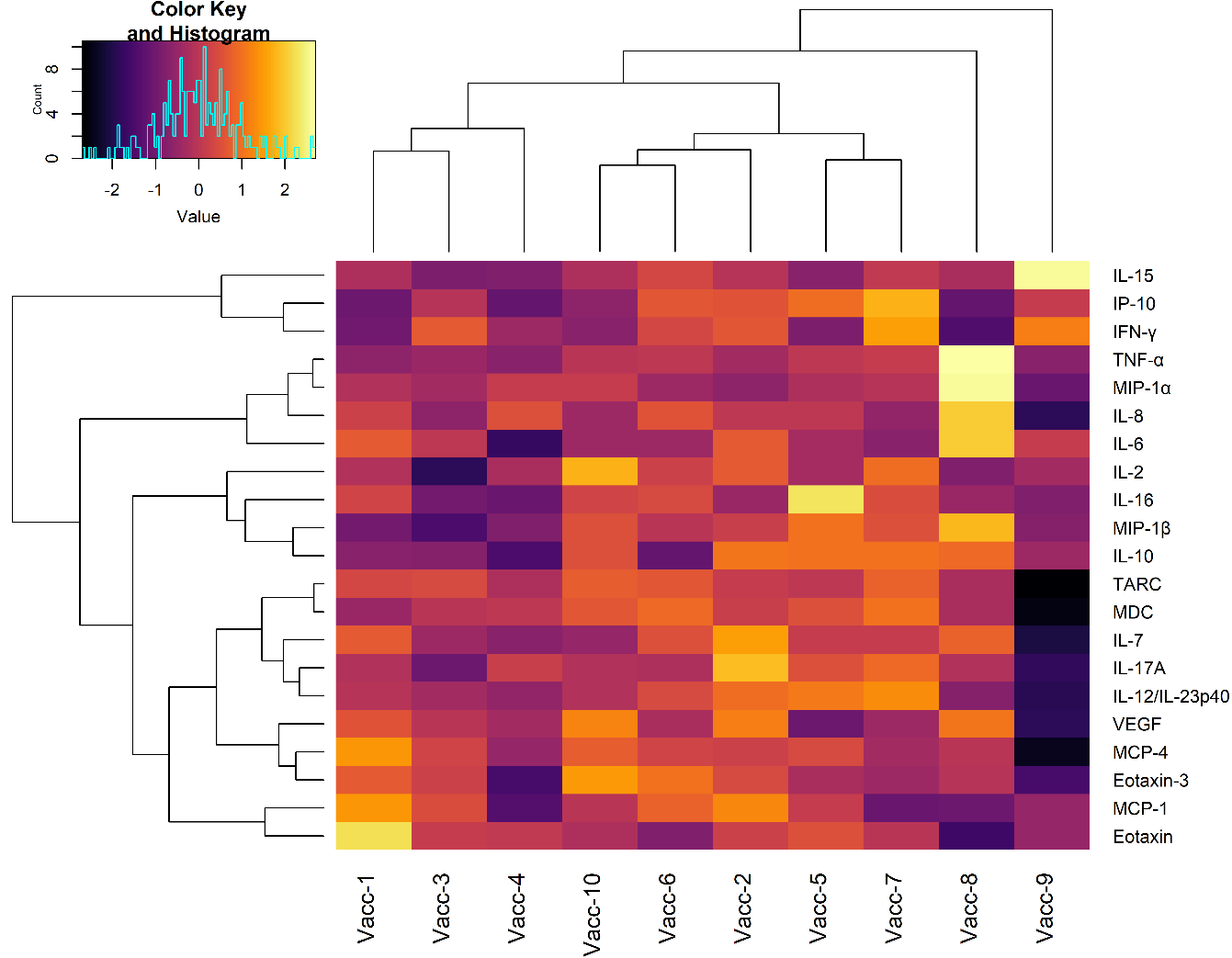
**

**Heatmap with unsupervised clustering of cytokine levels at baseline. a** Vaccinated patients (1-10), unvaccinated reference cohort (51-55), patients with smoldering multiple myeloma (SMM) and healthy donors (HD). **b** Vaccinated patients only.

**Supplementary figure 7**

**
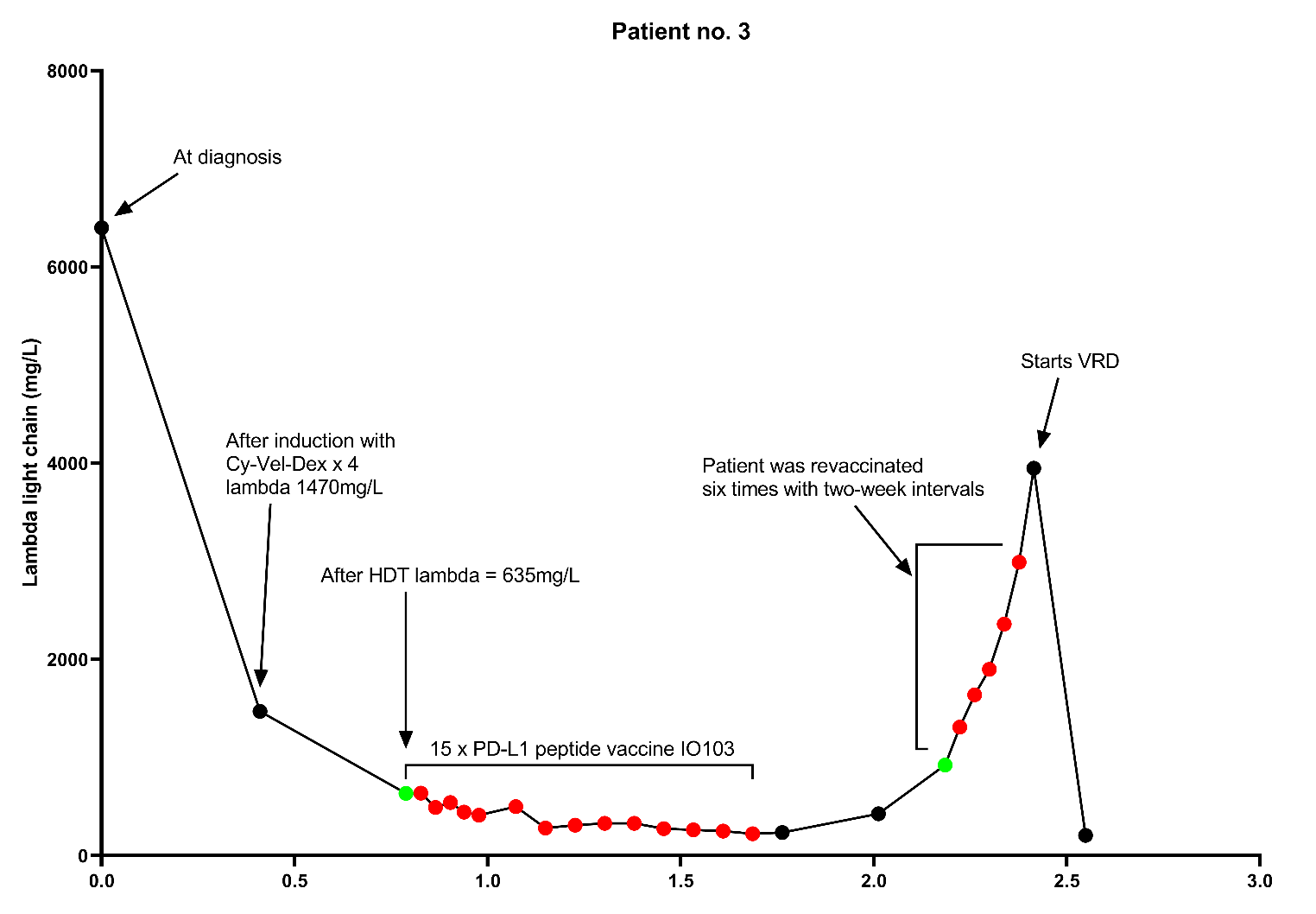
**

**M-component course in patient 3**

**Supplementary figure 8**

**a**

**
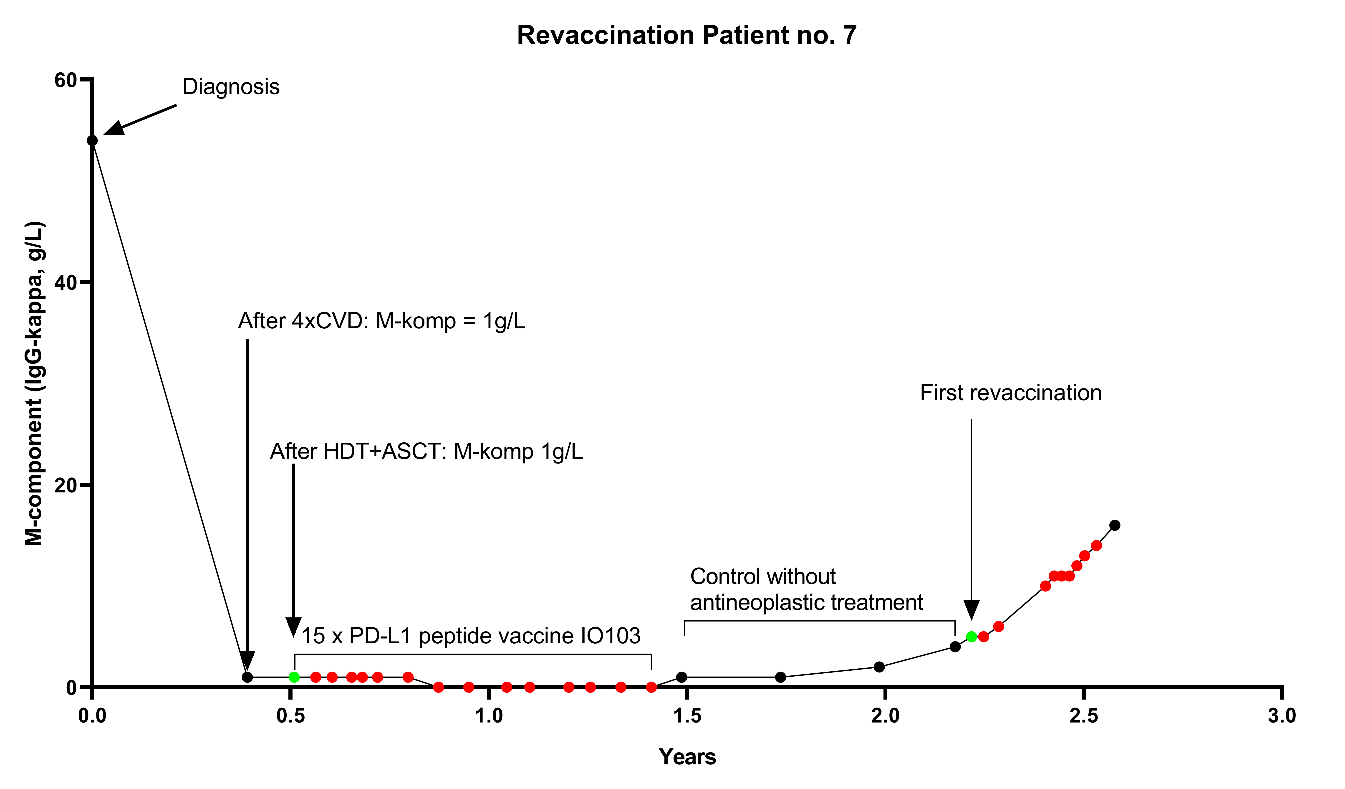
**

**b**

**
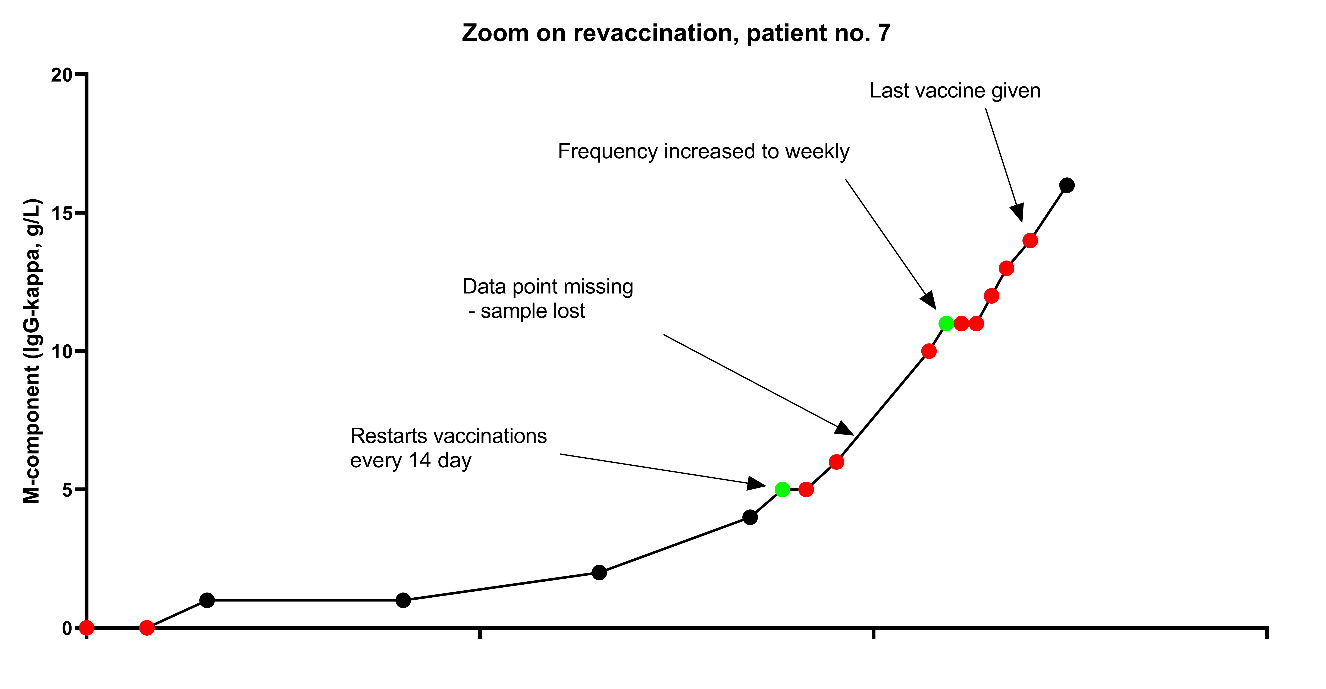
**

**M-component course in patient 7.**

**Supplementary figure 9**

**
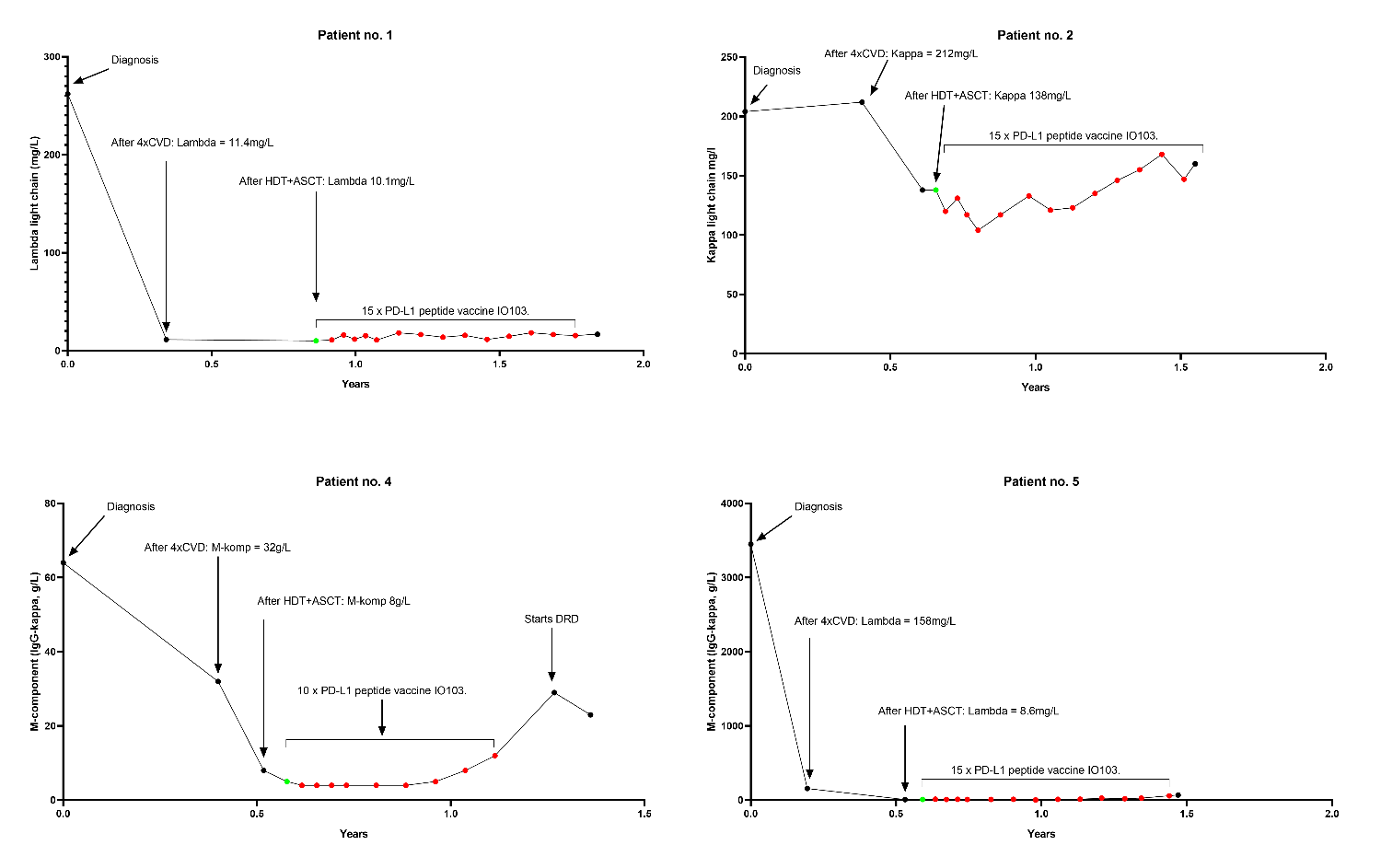
**

**Supplementary figure 9 (cont.)**

**
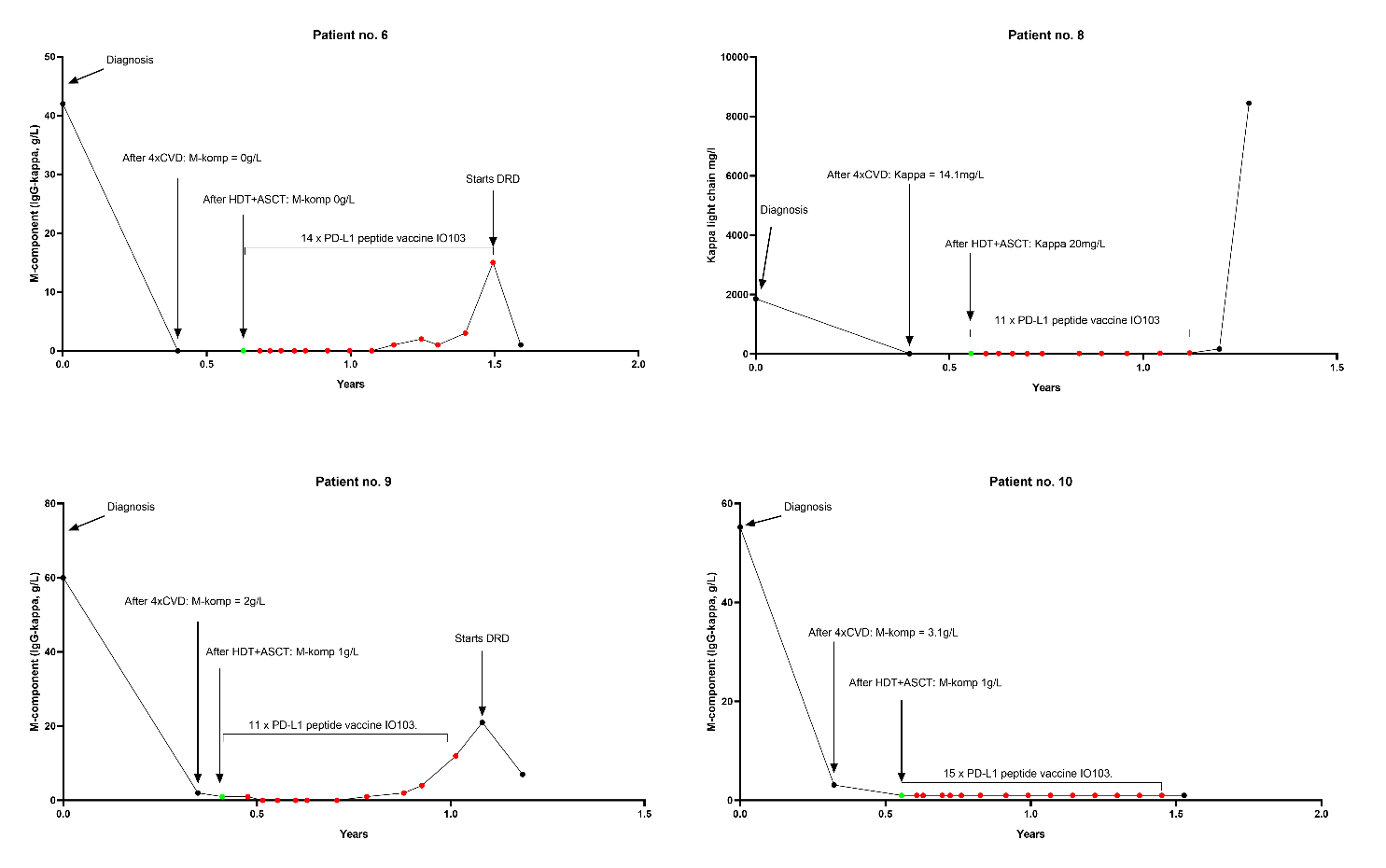
**

**M-component course in the patients who were not revaccinated.**

**Supplementary figure 10**

**
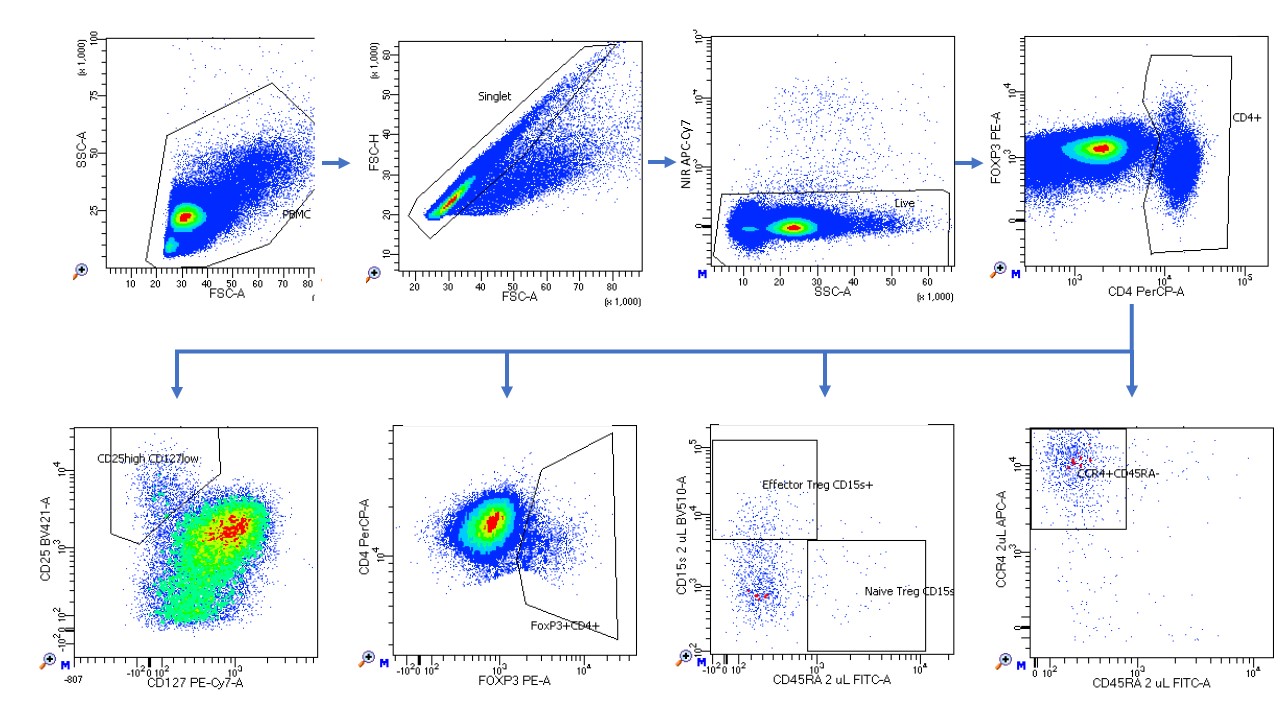
**

**Gating strategy for regulatory T cells (Treg).** A gate was set for PBMCs in the FSC/SSC plot. Subsequently doublets and dead cells were gated out. A CD4 gate was made to further gate on CD25+CD127- cells, FoxP3+, CD15s+ and CCR4+.

**Supplementary figure 11**

**
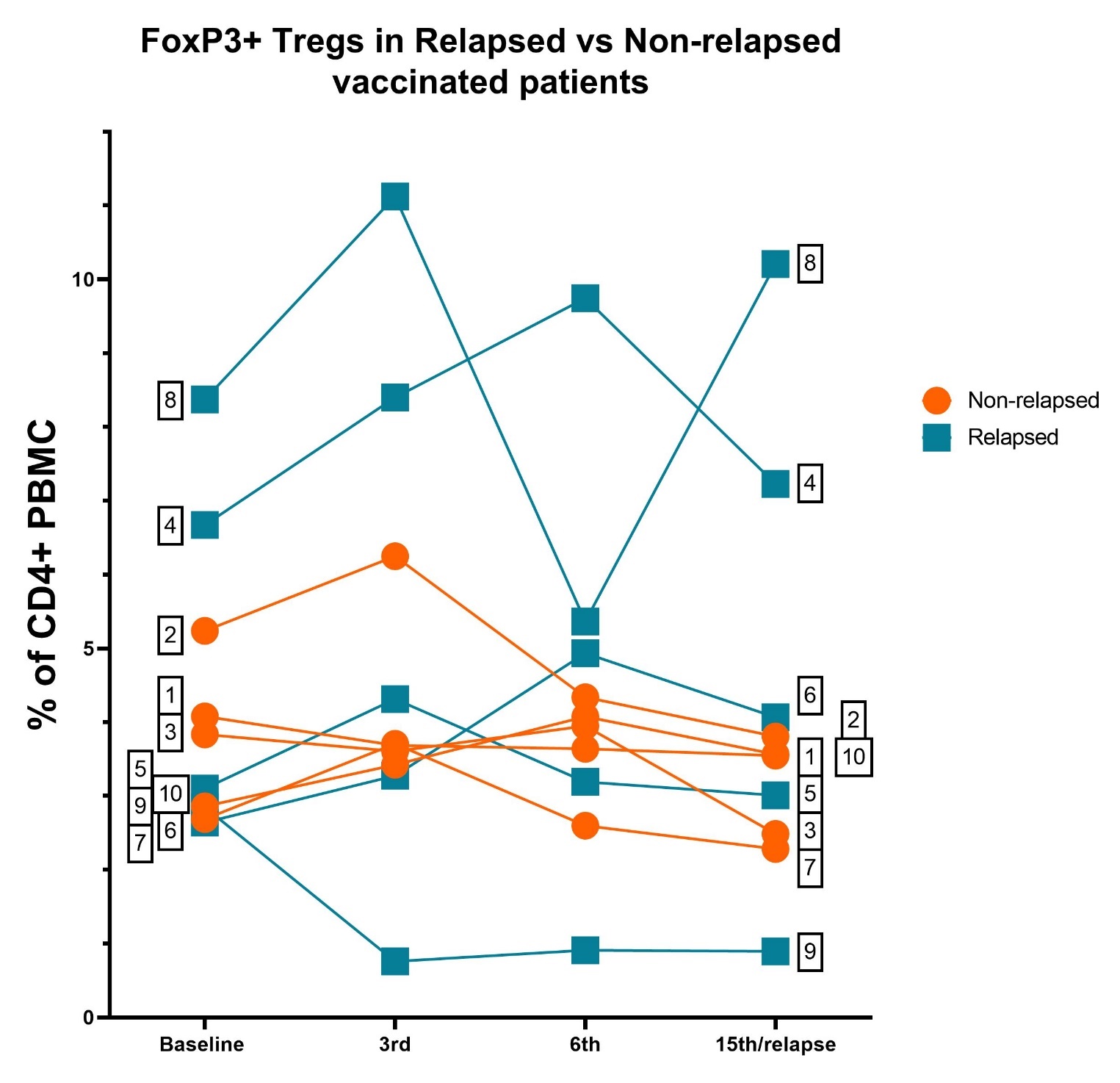
**

**FoxP3+ Tregs in individual patients.** Numbers refer to patient number.

**Supplementary figure 12**

**
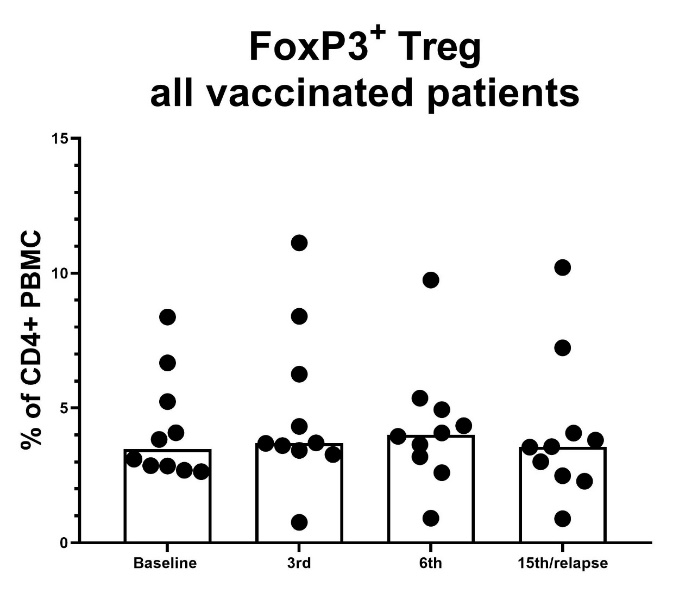
**

**FoxP3^+^ Treg in all vaccinated patients.**

**Supplementary figure 13**


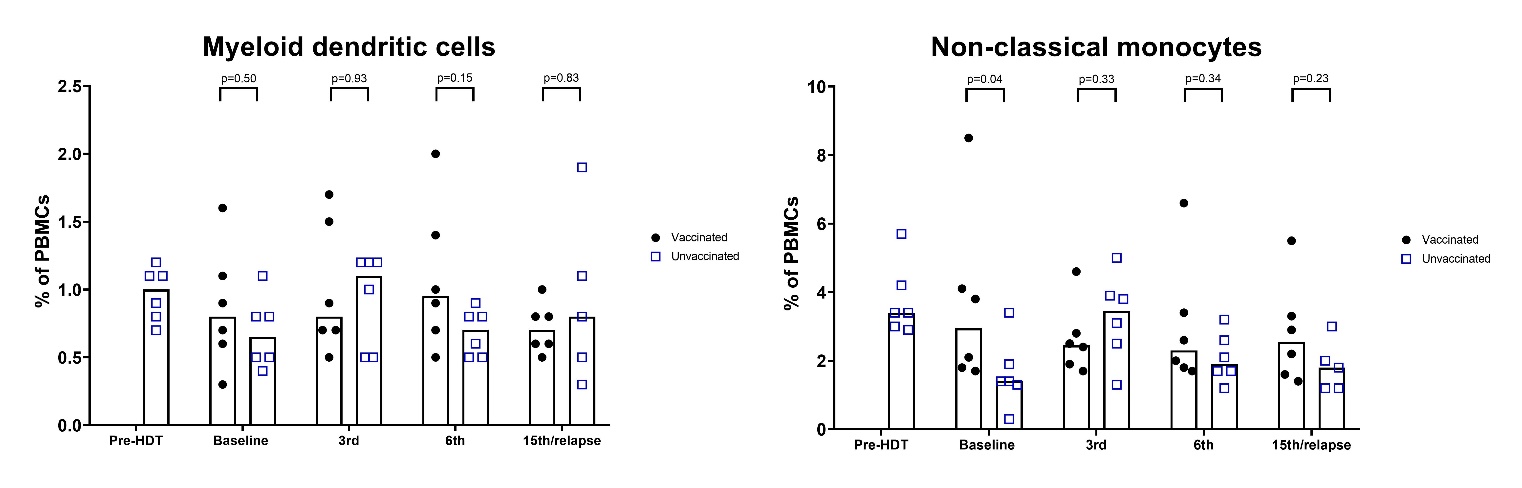


**Flowcytometric analysis of frequencies of dendritic cells.**
